# Inhibitors of the 20S proteasome β5 subunit as potent and selective agents against *Trichomonas vaginalis*

**DOI:** 10.1128/aac.00893-25

**Published:** 2025-11-11

**Authors:** Yukiko Miyamoto, Brianna M. Hurysz, Pavla Fajtova, Peter Eckmann, Mateus Sá Magalhães Serafim, Thales Kronenberger, Momen Al-Hindy, Patrick M. Dekker, Elmer Maurits, Herman S. Overkleeft, B. Mikael Bergdahl, Jehad Almaliti, William H. Gerwick, Anthony J. O’Donoghue, Lars Eckmann

**Affiliations:** 1 Department of Medicine, University of California San Diego 8784, La Jolla, California, USA; 2 Skaggs School of Pharmacy and Pharmaceutical Sciences, University of California San Diego 8784, La Jolla, California, USA; 3 Center for Discovery and Innovation in Parasitic Diseases, University of California San Diego 8784, La Jolla, California, USA; 4 Department of Computer Science and Engineering, University of California San Diego 8784, La Jolla, California, USA; 5 Department of Microbiology, Federal University of Minas Gerais (UFMG) 28114, Belo Horizonte, Minas Gerais, Brazil; 6 Institut für Medizinische Mikrobiologie und Hygiene, Partner-site Tübingen, German Center for Infection Research (DZIF), Tübingen, Germany; 7 Scripps Institution of Oceanography, University of California San Diego 8784, La Jolla, California, USA; 8 Gorlaeus Laboratories, Leiden Institute of Chemistry 201229, Leiden, the Netherlands; 9 Department of Chemistry and Biochemistry, San Diego State University 7117, San Diego, California, USA; The Children's Hospital of Philadelphia, Philadelphia, Pennsylvania, USA

**Keywords:** trichomoniasis, antimicrobials, proteasome subunits, docking simulations

## Abstract

*Trichomonas vaginalis* is the causative agent of the most prevalent, non-viral sexually transmitted infectious disease, yet treatment options are limited to three nitroheterocyclic antimicrobials of the same drug class, and resistance to these agents is a manifest clinical problem. The 20S proteasome is a validated new drug target against *T. vaginalis,* but inhibitors of the proteasome complex have so far only shown modest selectivity over human cells. We screened a library of 373 diverse peptide inhibitors with different reactive warheads against diverse *T. vaginalis* strains in a growth and survival assay and identified several compounds with potencies in the 10–20 nM range. Notably, these compounds were up to 200-fold selective for *T. vaginalis* over mammalian cells and could overcome metronidazole resistance. Removal of the epoxide or the adjacent methyl group in the inhibitors carrying an epoxyketone functionality abolished activity, underlining the functional importance of this warhead. Biochemical and whole-cell assays of inhibitory activity against each of the three catalytically active subunits (β1, β2, and β5) of the proteasome showed that inhibition of β5 was sufficient to mediate activity against *T. vaginalis*. Furthermore, the high selectivity of the best compounds came from their preferential inhibition of *T. vaginalis* β5 over human β5. Docking calculations of the most selective inhibitor to the *T. vaginalis* substrate-binding pocket revealed the formation of two stabilizing hydrogen bonds with a threonine residue that is absent in the human proteasome. These results encourage the therapeutic development of highly potent and selective proteasome inhibitors against *T. vaginalis*.

## INTRODUCTION

The amitochondriate protozoan pathogen, *Trichomonas vaginalis*, is the causative agent of the most prevalent, non-viral sexually transmitted infectious disease, with an estimated 5–7 million cases in the U.S. and >200 million in the world each year (1). Approximately 3% of U.S. women of reproductive age and as many as 16% in other countries are infected with *T. vaginalis* (1, 2). In addition to infections of the urogenital tract, trichomoniasis increases the risk of adverse pregnancy outcomes (3), HIV transmission (4), and the incidence and severity of cervical and prostate cancers (5, 6). Given its prevalence, its association with multiple diseases, and the increase in drug resistance (7), the development of new antimicrobials against trichomoniasis is an urgent need, particularly in women, where infection can persist for months or even years, compared to generally less than 10 days in men (8).

Only three drugs of the same class are FDA approved for the treatment of trichomoniasis: the nitroheterocyclic drugs metronidazole, tinidazole, and secnidazole. Oral drug administration leads to clinical and microbiological cure in the majority of cases, but treatment failures occur in a significant fraction of patients, ranging from 1% to 17% depending on the population sampled (9, 10). A U.S. survey found that 4.3% of 538 isolates of *T. vaginalis* showed metronidazole resistance (11), and cross-resistance is common among the three heterocyclic drugs. In addition, while generally safe and effective, the current therapies have significant liabilities, as exemplified by the moderate to severe adverse effects of metronidazole, which include neurologic maladies (peripheral neuropathy, cerebellar syndrome, encephalopathy, and meningitis), and intolerable nausea and gastric cramping (12). Patient compliance is of concern due to seemingly benign but common adverse effects, such as metallic taste, nausea, dizziness, and alcohol intolerance (13).

The proteasome is a large (20S) multi-subunit protein complex that regulates numerous cellular functions, including protein turnover and degradation of misfolded proteins (14). In eukaryotes, proteins destined for degradation are first conjugated with one or more ubiquitin chains to lysine residues. These ubiquitinated proteins are then recognized and unfolded by regulatory proteins that flank the proteolytic core of the proteasome. Once the protein substrate has engaged the proteasome, the ubiquitin chains are removed by deubiquitinases (15). The unfolded protein substrates are threaded into the barrel-shaped proteolytic core and degraded into short peptide sequences, while the released ubiquitin chains are recycled to label new substrates. The 20S core of the proteasome is formed by two stacked rings of seven β subunits sandwiched between two rings of seven α subunits. Only the β1, β2, and β5 subunits have hydrolytic activity, and each has a distinct substrate specificity. In humans, all cells express the 14 subunits that make up the constitutive proteasome (c20S). Immune cells express three additional catalytically active proteasome subunits, β1i, β2i, and β5i, with “i” denoting the inducible or immune cell-derived forms, which replace β1, β2, and β5, thereby forming the immunoproteasome (i20S) (16).

The genomes of eukaryotic pathogens encode proteasome subunits that are structurally and functionally similar to the mammalian subunits but have different enzymatic specificities (17). Consequently, proteasome inhibitors can be developed that are selective for pathogen proteasomes over the human proteasome. Such selective inhibitors have shown promise in preclinical studies for the treatment of leishmaniasis (18), malaria (19), schistosomiasis (20), and babesiosis (21). For *T. vaginalis*, our earlier studies with peptide inhibitors validated the 20S proteasome as an antimicrobial target, but the selectivity of the inhibitors over human cells was only modest (<10-fold) (22). Therefore, in the current study, we have screened a large library of proteasome inhibitors for activity against *T. vaginalis* and have explored the mechanistic basis of improved selectivity with *in vitro* proteasome assays and structural modeling.

## RESULTS

### Library screen for identification of proteasome inhibitors with high potency and selectivity toward *T. vaginalis*

**Fig 1:**
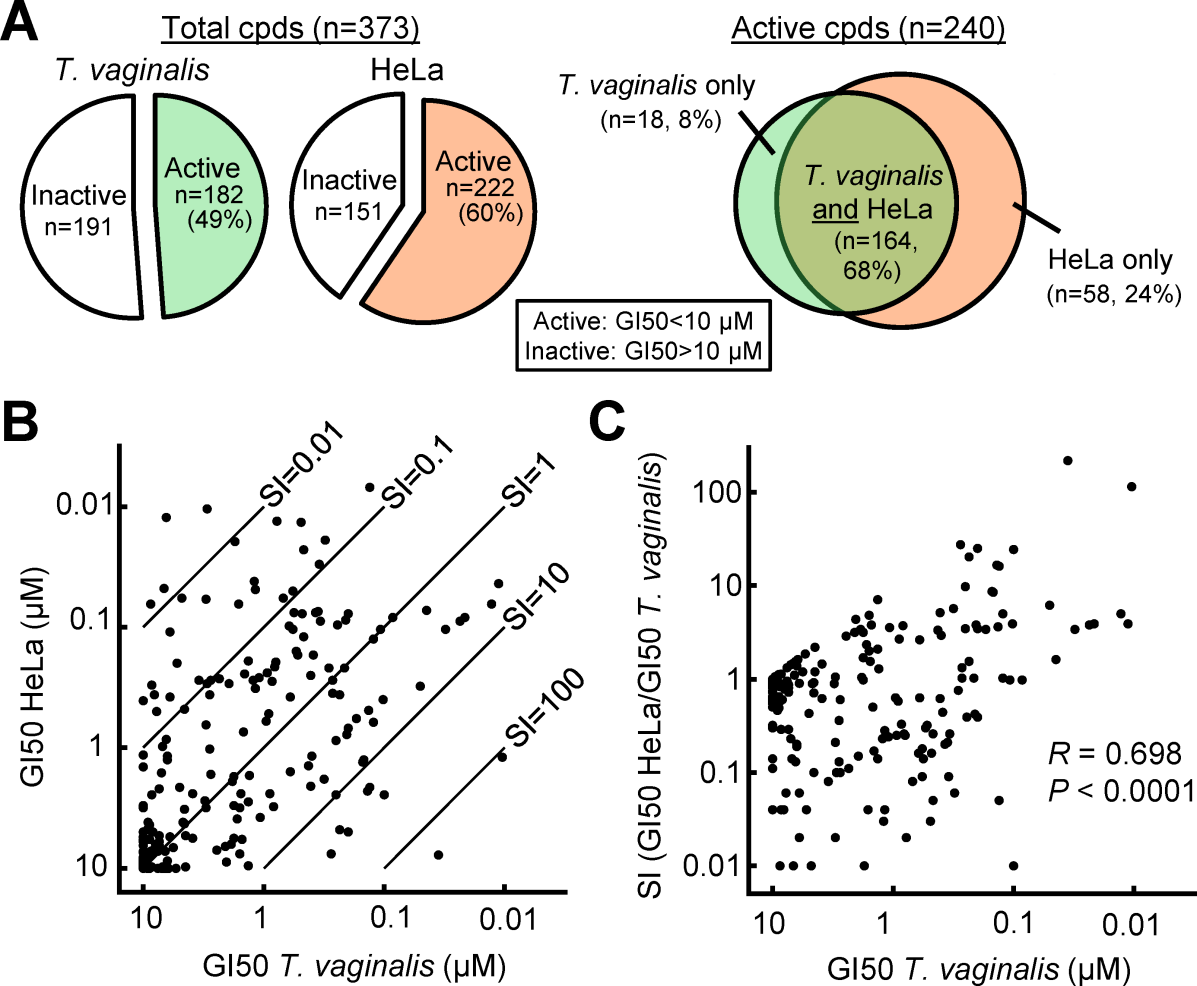
Screening of proteasome inhibitor library for activity against *T. vaginalis* and human cells. (**A**) A library of 373 proteasome inhibitors was assayed for inhibition of growth and survival (growth inhibition 50%, GI50) against *T. vaginalis* strain F1623 and the human cell line HeLa. The number of active compounds (GI50 <10 µM) is indicated by the colored areas (green, *T. vaginalis*; orange, HeLa). (**B**) Correlation of GI50 values of individual compounds against *T. vaginalis* F1623 and HeLa cells. Diagonal lines represent the indicated selectivity indices (SI; ratio of GI50 in HeLa cells over GI50 in *T. vaginalis*). (**C**) Correlation of activity (GI50) against *T. vaginalis* and SI values for individual compounds. Pearson’s correlation coefficient (*R*) and probability (*P*) are shown. Each data point in (**B**) and (**C**) represents the average of at least three independent experiments. Detailed data for all compounds are shown in Table S1.

Prompted by our earlier findings with a small number of proteasome inhibitors that showed sub-micromolar (200–500 nM) activity against *T. vaginalis* and modest selectivity (up to 10-fold) over human cells (22), we assembled a greatly expanded library of 373 diverse proteasome inhibitors, consisting mostly of N-capped tripeptides coupled at the C-terminal end with a reactive functional group, including epoxyketone, boronic acid, or vinyl sulfone functional groups (Table S1). The library was evaluated for activities against a representative *T. vaginalis* strain, F1623, in a 24 h growth and survival assay (23). We found that 49% of compounds in the library were active against the parasite, as determined by a 50% growth inhibitory concentration (GI50) of <10 µM (Fig. 1A; Table S1). By comparison, testing of the same compounds against human cells (HeLa) revealed a slightly higher percentage of active compounds (60%). Furthermore, the majority of active compounds were effective against both *T. vaginalis* and HeLa cells, while 24% of active compounds were found to have activity only in HeLa cells, and only 8% of active compounds were solely active in *T. vaginalis*. These findings are in line with the fact that many of the compounds were originally developed as potential inhibitors of the human proteasome (24). Comparison of the GI50 values revealed that the activities against the parasite and human cells were not tightly correlated, as illustrated by the finding that several compounds had high activity against *T. vaginalis* but not against human cells while others showed the reverse pattern (Fig. 1B). Consequently, the selectivity index (SI), calculated as GI50 HeLa/GI50 *T. vaginalis*, ranged from >100, indicative of a strong preference for *T. vaginalis*, to <0.01, which indicates preferential activity against human cells (Fig. 1B). While the most potent *T. vaginalis* inhibitors tended to also have high selectivity, only modest correlation was observed between potency and selectivity across all library compounds (Fig. 1C). Together, when compared to our prior findings (22), the library screen has identified several new proteasome inhibitors with marked (up to 50-fold) improvements in potency against *T. vaginalis* and >200-fold selectivity over human cells.

### SAR analysis of proteasome inhibitory activity against *T. vaginalis*

**Fig 2:**
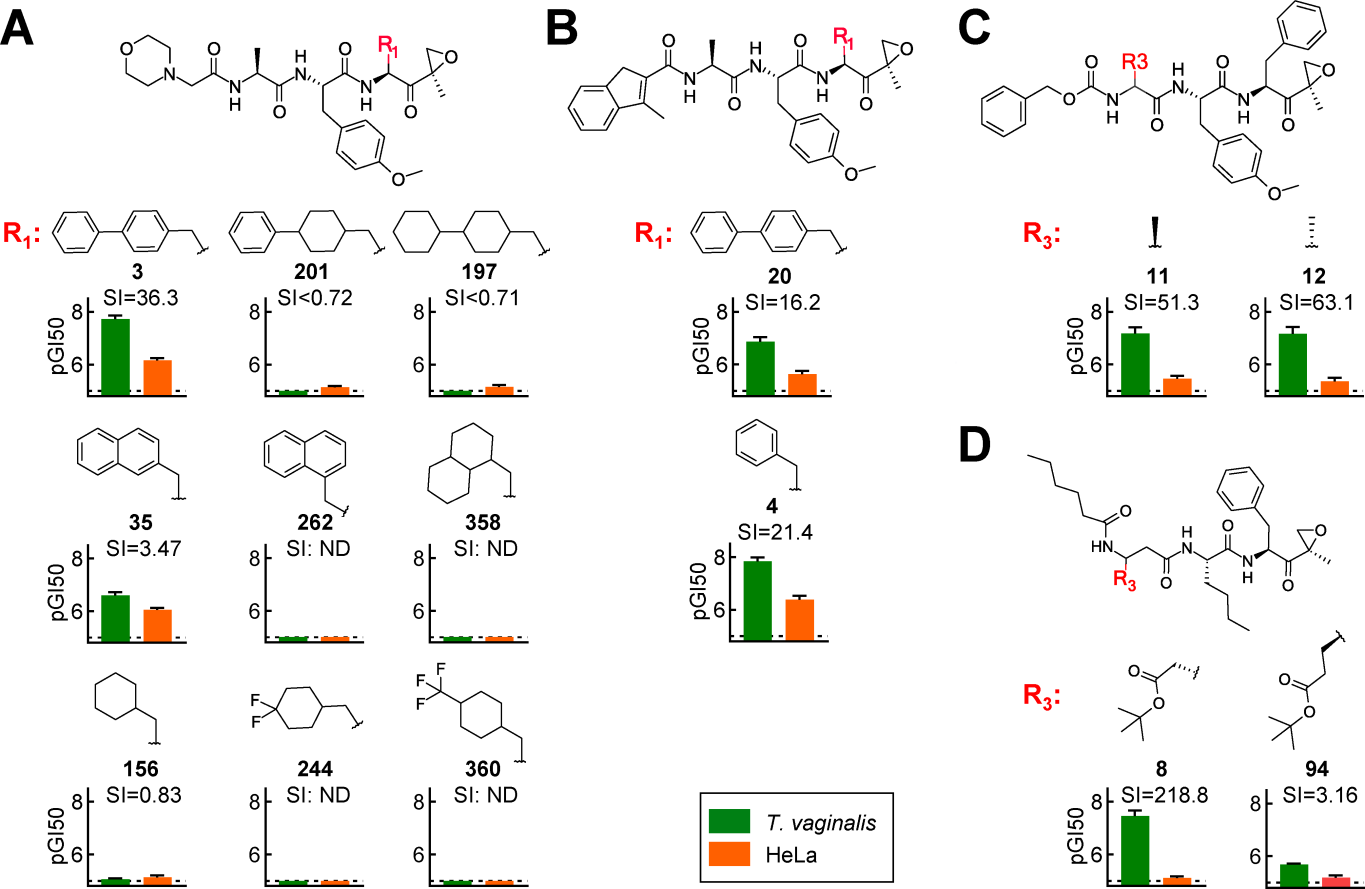
Analysis of SAR of library compounds. The growth inhibitory activity (pGI50) of the depicted compounds against *T. vaginalis* F1623 (green bars) and human HeLa cells (orange bars) was determined in growth and survival assays. Results are shown as mean ± standard error of at least three independent experiments. The SI was calculated as the ratio of GI50 in HeLa cells over *T. vaginalis*. The dashed lines indicate assay sensitivity. SAR analysis was performed by examining modifications at different positions among the existing library compounds. (**A**) Influence of the depicted singular R_1_ modifications in the P1 position of the shown tripeptide-epoxyketones with identical P2 and P3 residues and a morpholino N-cap. (**B**) Impact of the depicted R_1_ modifications in the P1 position of the shown tripeptide-epoxyketones with a methylindenyl N-cap. (**C**) Influence of the stereochemical configuration of the methyl group in the P3 position in the depicted tripeptide-epoxyketones with a carboxybenzyl N-cap. (**D**) Impact of the depicted R_3_ modifications in the P3 position of the shown tripeptide-epoxyketones with a pentyl N-cap. Bolded compound numbers correspond to those listed in Table S1. ND, not determined.

For the N-capped tripeptide-epoxyketones, the library had sufficient structural diversity and density to allow for meaningful preliminary structure-activity relationship (SAR) analysis. For P1, an extended aromatic structure (biphenyl) with moderate rotational flexibility displayed excellent potency and selectivity, while ring saturation (e.g., bicyclohexyl) or rigid aromatic structures (e.g., naphthyls) abolished activity or selectivity (Fig. 2A). Similarly, a phenyl in the P1 position was also associated with good selectivity and even greater potency (Fig. 2B and D). For the N-cap (P4), a variety of different groups, including morpholino, methylindenyl, carboxybenzyl, and pentyl, were present in highly active and selective compounds (Fig. 2A through D), suggesting that the identity of the N-cap was not critical for selective activity against *T. vaginalis*. For P2, the library only had compounds with one of two amino acids, O-Methyl-L-Tyr (Fig. 2A through C) or L-norleucine (Fig. 2D), both of which were associated with highly potent and selective compounds. For P3, many of the active compounds had either L-Ala or D-Ala at this site (Fig. 2C), suggesting that the stereochemical orientation of the methyl group was not critical. We also had compounds with *tert*-butyl-protected β-Asp in P3 with excellent activity and an SI of 219, the highest selectivity of all tested compounds, while replacement of the protected β-Asp with a *tert*-butyl-protected β-Glu largely abolished activity and selectivity (Fig. 2D). These data indicate that the P3 position can be occupied by residues larger than alanine without compromising anti-parasitic activity, but subtle side chain differences can impact activity and selectivity.

**Fig 3:**
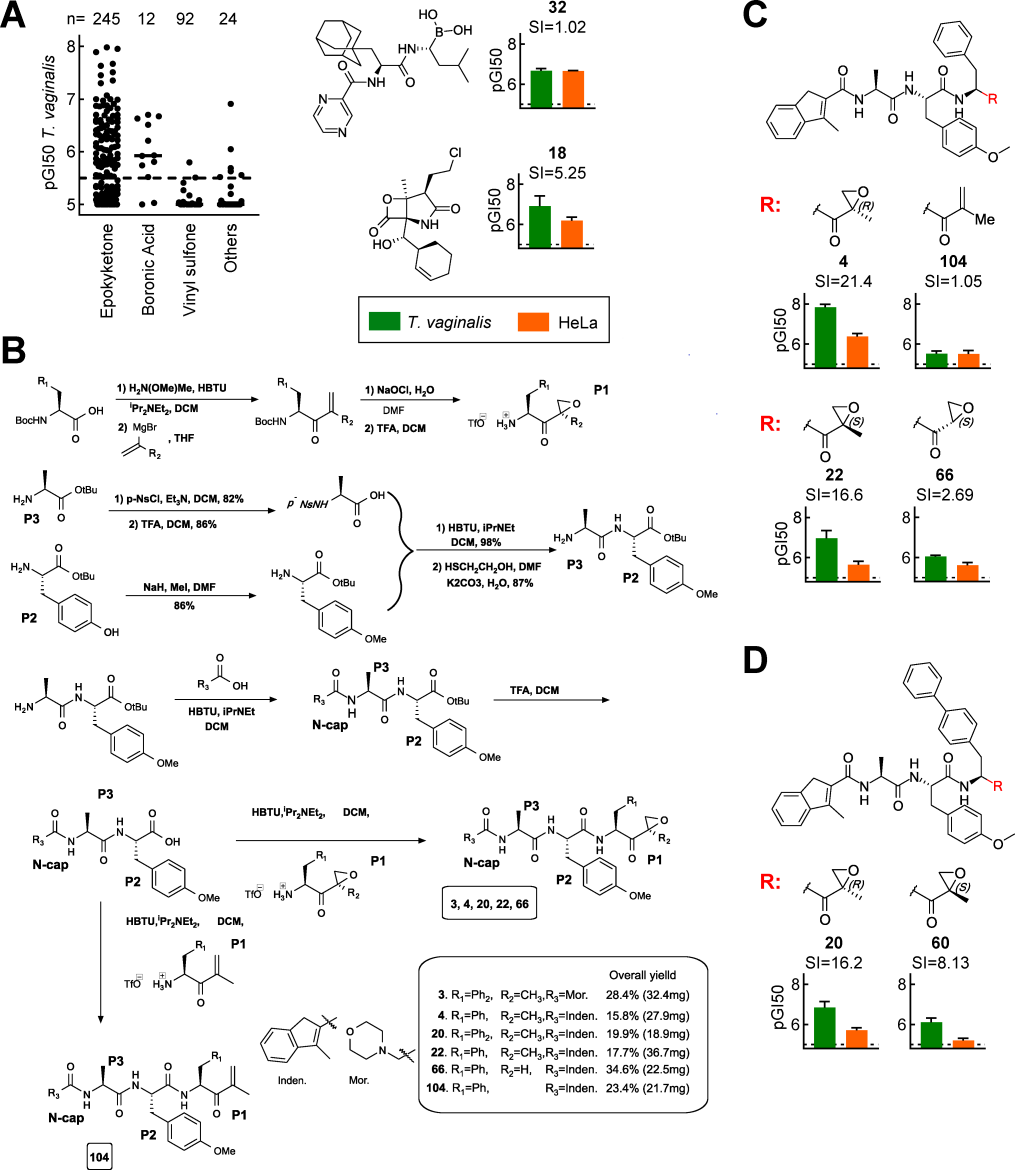
Importance of C-terminal warhead for proteasome inhibitory activity in *T. vaginalis*. (**A**) Activity (pGI50) of proteasome inhibitors against *T. vaginalis* F1623, categorized by their indicated warheads. Each data point represents the mean value of at least separate experiments. Two representative compounds other than epoxyketones are shown on the right with their activities against *T. vaginalis* (green bars) and human HeLa cells (orange bars), as well as their SI. (**B**) Schematic representation of the strategy for synthesizing new N-capped tripeptide proteasome inhibitors with modifications of the C-terminal warhead. (**C, D**) The activities of the depicted inhibitors against *T. vaginalis* F1623 (green bars) and human HeLa cells (orange bars) are shown, along with their SI values. Bold compound numbers correspond to those listed in Table S1. All bar graphs in panels **A**, **C**, and **D** show mean ± SEM of at least three independent experiments. The dashed lines indicate assay sensitivity.

Our library contained compounds with various reactive warheads that directly target the catalytic Thr residue of the parasite and human proteasomes. While compounds with epoxyketone warheads were the most common and active, several compounds with boronic acid and vinyl sulfone warheads also exhibited significant activity against *T. vaginalis* (Fig. 3A). However, none of these alternate warhead compounds showed meaningful selectivity (Table S1), most likely due to their peptide backbone being sub-optimal for selective binding to the *T. vaginalis* proteasome. Consequently, we concentrated subsequently on the epoxyketone compounds.

To explore the importance of the epoxyketone warhead, we synthesized several new derivatives of active compounds with modifications of the warhead (Fig. 3B). A compound in which the epoxide of the active parental compound (**4**) was replaced with an electron-deficient alkene was only minimally inhibitory for *T. vaginalis* and human cells (**104**, Fig. 3C), indicating that the reactive epoxide was critical for activity. To explore the importance of the stereochemical configuration of the epoxyketone warhead, we synthesized a stereoisomer of **4** in which the epoxide-adjacent methyl group was in the *S* configuration (**22**), rather than the *R* configuration. The *S* stereoisomer had good selectivity but lower potency against *T. vaginalis* compared to the *R* stereoisomer (Fig. 3C). We confirmed this stereochemical finding for another inhibitor that carried a biphenyl (rather than phenyl) in the P1 position. Here too, the *S* stereoisomer (**60**) was overall less potent than the *R* stereoisomer (20) but retained much of its selectivity for the parasite over human cells (Fig. 3D). Finally, to assess the impact of the methyl group adjacent to the epoxide ring, we synthesized a compound (**66**) lacking this methyl substituent. Similar to the epoxide removal, absence of the adjacent methyl group led to a marked (~50-fold) reduction in activity against *T. vaginalis* and (~10-fold) loss in selectivity (Fig. 3C). Together, these data indicate that the reactive epoxide and adjacent methyl group are important for both anti-parasitic activity and selectivity, while the stereochemical configuration of the methyl group is primarily relevant for overall potency but less so for selectivity.

### Activity of proteasome inhibitors against diverse and metronidazole-resistant *T. vaginalis* strains

**Fig 4:**
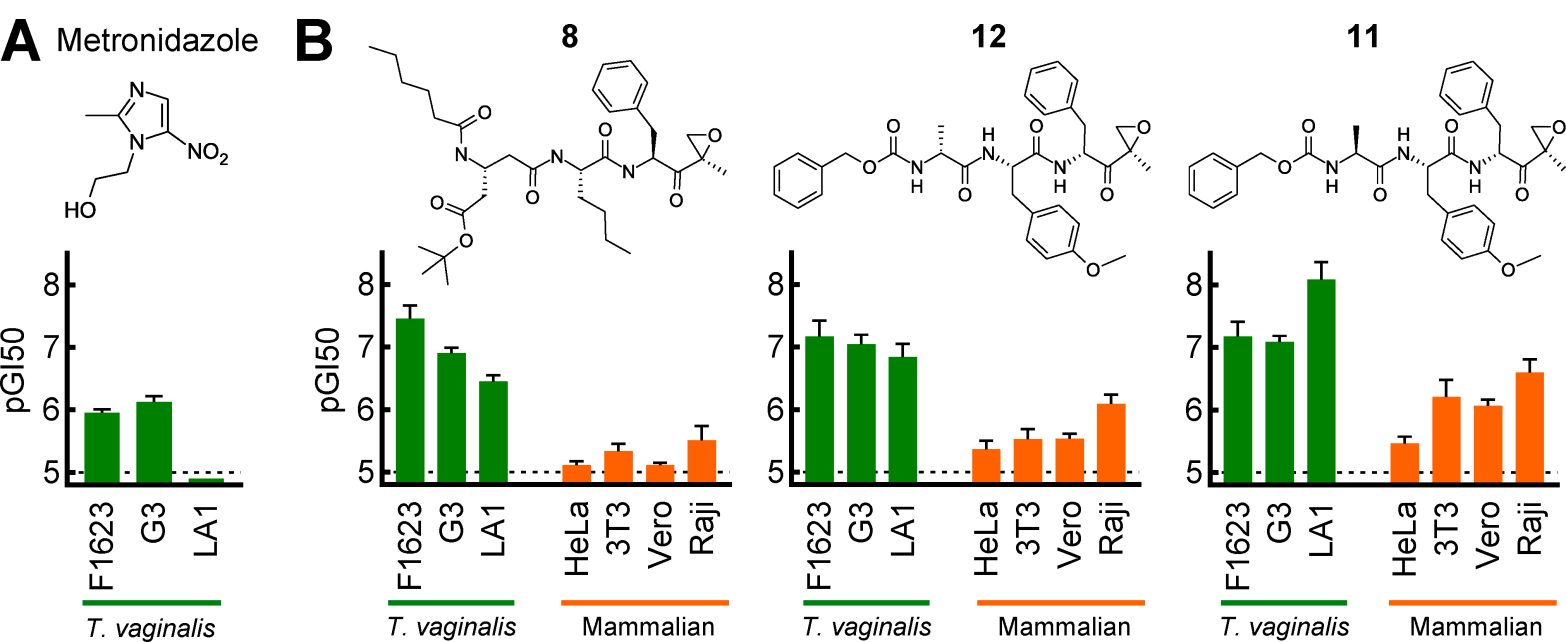
Activity of proteasome inhibitors against diverse *T. vaginalis* strains and mammalian cell lines. (**A**) Activity (pGI50) of metronidazole against the indicated *T. vaginalis* strains. LA1 is a metronidazole-resistant strain. (**B**) The depicted proteasome inhibitors were assayed for inhibition of growth and survival (pGI50) against the indicated *T. vaginalis* strains (green bars) and mammalian cell lines (orange bars). All results are shown as mean ± SEM of at least three independent experiments. Bold compound numbers correspond to those listed in Table S1. The dashed lines indicate the assay sensitivity.

Clinical isolates of *T. vaginalis* exhibit substantial diversity in their responses to antimicrobial agents, and a significant proportion of strains exhibit resistance to the only clinically used trichomonacidal drugs, metronidazole, tinidazole, and secnidazole (7, 10, 11). To test the broader utility of proteasome inhibitors as anti-trichomonal agents, we tested three of the most promising inhibitors against several independently established clinical *T. vaginalis* strains, including strains from patients who failed metronidazole therapy and exhibited resistance to metronidazole *in vitro* (25). All three inhibitors displayed excellent activity against the three diverse *T. vaginalis* strains, although strain-specific differences were observed between the inhibitors (Fig. 4). Importantly, the inhibitors were active against a metronidazole-resistant *T. vaginalis* strain (LA1, Fig. 4A), indicating that the resistance mechanism against metronidazole had no impact on the proteasome inhibitor activity. We also evaluated the compounds for cytotoxicity against several mammalian cells, including the human HeLa cells used for the initial screens, two non-transformed cell lines (3T3, Vero), and a human B cell line (Raji). While all three inhibitors exhibited some cytotoxicity against the mammalian cells, they showed 10- to 100-fold greater potency against *T. vaginalis* (Fig. 4B). Of note, the cytotoxicity was greatest against human Raji B lymphocytic cells, which may relate to the original goal for many of the compounds in the library to target the immunoproteasome in lymphocytes (26). Together, these findings indicate that the most promising proteasome inhibitors are broadly active against different *T. vaginalis* strains, can overcome metronidazole resistance, and show consistent selectivity over mammalian cells.

### Differential subunit specificity and selectivity of proteasome inhibitors

**Fig 5:**
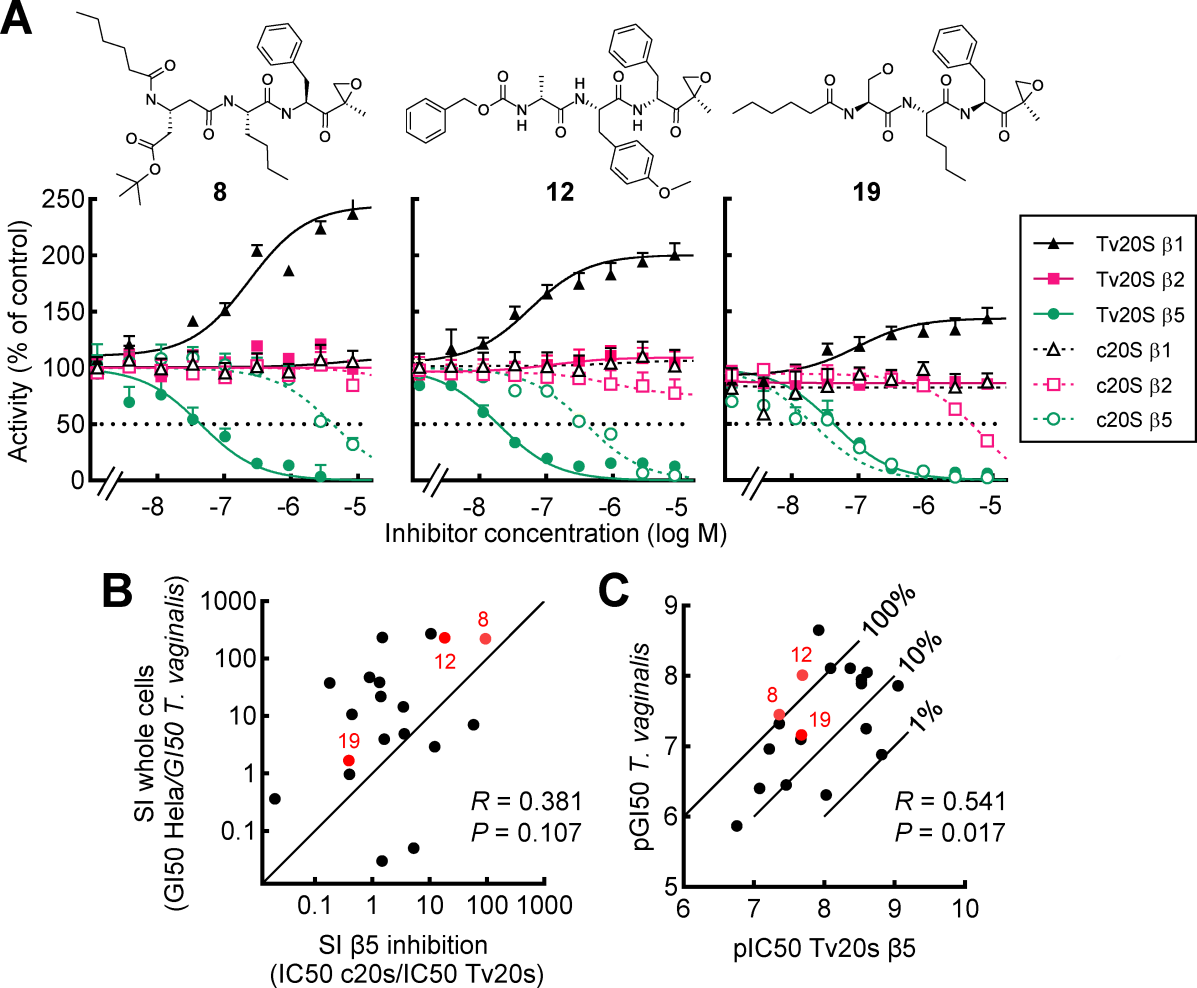
*In vitro* selectivity of proteasome inhibitors against *T. vaginalis* proteasome subunits. (**A**) Activity of the indicated proteasome inhibitors (bold numbers, structures shown in Fig. 4) against the β1 (black), β2 (red), and β5 (green) catalytic subunits of the purified proteasomes of *T. vaginalis* (Tv20S, closed symbols and solid lines) and human cells (c20S, open symbols and dashed lines) was assayed *in vitro* using subunit-specific fluorescent reporter substrates. Proteasomes were added to reporter substrates and inhibitors, and enzyme activity was measured using a fluorescence plate reader for the next 3 h. Activity is expressed as a percentage relative to control proteasome preparations without added inhibitor. Results are shown as mean ± SEM of three independent experiments. (**B**) The SI for β5 inhibition was calculated as the ratio of the IC50 values for Tv20S β5 over c20S β5 (Table 1). SI for growth inhibition of whole cells was calculated as the ratio of GI50 in HeLa cells over *T. vaginalis* (Table S1). The SI values for β5 inhibition *in vitro* and growth inhibition in whole cells were then plotted for the selected 20 inhibitors. The diagonal line represents 1:1 ratios of SI values *in vitro* and in whole cells. (**C**) Correlation between Tv20S β5 inhibition *in vitro* (pIC50) and growth inhibition in live *T. vaginalis* (pGI50). Diagonal lines show the indicated percentages of whole cell activity relative to β5 inhibitory activity *in vitro*. For panels B and C, each data point represents the mean values of three independent experiments for one inhibitor. Pearson’s correlation coefficient (*R*) and probability (*P*) are shown in the graphs. The β- subunit inhibition profiles for the three inhibitors highlighted in red in **B** and **C** are shown in panel **A**.

Eukaryotic proteasomes have three proteolytically active subunits, β1, β2, and β5, each with a distinct preference for the protein sequence that is cleaved. To determine which of these subunits, alone or in combination, is responsible for the observed activities against *T. vaginalis*, we conducted enzymatic assays with purified recombinant *T. vaginalis* 20S (Tv20S) proteasome (27), using three fluorescent reporter substrates that each have a strong preference for cleavage by only one of the three active subunits of Tv20S (28). We selected 20 inhibitors that represented a wide selectivity range in whole cells. All inhibitors inactivated the Tv20S β5 subunit with IC50 values ranging from 0.88 to 172 nM (Table 1; Fig. 5A). None of the newly identified inhibitors inactivated the β1 subunit, and only two inhibitors inhibited the β2 subunit, but only at very high concentrations (Table 1). We also tested three control inhibitors (bortezomib, carmaphycin B, and marizomib) with known broad activities against several proteasome subunits (29). All three were active against β5 while also targeting β1 and/or β2 at higher concentrations (Table 1). Interestingly, for all compounds that preferentially targeted the β5 subunit, a dose-dependent increase in activity of the β1 subunit was evident (Table 1; Fig. 5A). To assess the importance of the epoxyketone warhead for *in vitro* activity, we also evaluated the alkene derivative that lacked an epoxide (**104**) and that had lost activity against whole cells (see Fig. 3C above). As expected, this compound did not inhibit any of the three catalytic β subunits of Tv20S (Table 1), confirming that the active warhead is required for target inactivation. Together, these results show that inhibition of the Tv20S β5 subunit alone appears to be sufficient for mediating whole-cell activity against *T. vaginalis*.

**TABLE 1:** Catalytic subunit specificity of proteasome inhibitors^*a*^

Proteasome inhibitor	*T. vaginalis* Tv20S proteasome Inhibition of β subunits (IC50, nM)			Human c20S proteasome Inhibition of β subunits (IC50, nM)			β5 SI ratio (c20S/Tv20S)
	β1	β2	β5	β1	β2	β5	
	Mean ± SD	Mean ± SD	Mean ± SD	Mean ± SD	Mean ± SD	Mean ± SD	
8	Activation	No inhibition	43.7 ± 12.3	No inhibition	No inhibition	4,102 ± 851	93.87
75	Activation	No inhibition	172 ± 91.7	No inhibition	No inhibition	No inhibition	>72
12	Activation	No inhibition	20.6 ± 5.39	No inhibition	No inhibition	379 ± 61.2	18.40
45	Activation	No inhibition	82.6 ± 32.1	No inhibition	No inhibition	1,008 ± 200	12.20
11	Activation	No inhibition	4.29 ± 2.23	No inhibition	No inhibition	45.3 ± 4.67	10.56
38 (CPB)	Activation	440 ± 101	1.51 ± 0.69	895 ± 510	14.6 ± 0.83	7.92 ± 0.72	5.25
2	Activation	3,551 ± 1576	2.98 ± 0.66	No inhibition	4,081 ± 665	10.8 ± 0.82	3.62
91	Activation	No inhibition	2.52 ± 1.05	No inhibition	3,01.6 ± 39.8	8.81 ± 0.69	3.50
1	Activation	1594 ± 188	2.93 ± 0.57	No inhibition	1,131 ± 463	4.73 ± 0.23	1.61
3	Activation	No inhibition	12 ± 1.94	No inhibition	271 ± 26.4	18.1 ± 3.26	1.51
50	Activation	No inhibition	9.25 ± 1.81	No inhibition	64.7 ± 8.19	13.8 ± 4.21	1.49
22	Activation	No inhibition	60.5 ± 8.1	No inhibition	No inhibition	84.8 ± 20.2	1.40
4	Activation	No inhibition	8.21 ± 1.16	19738 ± 8582	1690 ± 410	11 ± 1.12	1.34
20	Activation	No inhibition	44.1 ± 6.71	No inhibition	No inhibition	39.4 ± 4.76	0.89
5	Activation	No inhibition	2.43 ± 0.39	No inhibition	68.2 ± 13	1.06 ± 0.08	0.44
100	Activation	No inhibition	34.4 ± 3.74	No inhibition	3,948 ± 447	13.7 ± 2.56	0.40
19	Activation	No inhibition	21 ± 2.47	No inhibition	2,163 ± 316	8.16 ± 1.8	0.39
18 (MZB)	403 ± 77	No inhibition	0.88 ± 0.11	66.5 ± 11.7	46.7 ± 11.2	0.16 ± 0.01	0.18
43 (BTZ)	68.1 ± 16	30.8 ± 19.9	21.6 ± 1.69	20.5 ± 1.81	23,136 ± 7,682	0.49 ± 0.18	0.02
104	No inhibition	No inhibition	No inhibition	No inhibition	No inhibition	No inhibition	ND

^*a*^ The indicated proteasome inhibitors were tested *in vitro* for inhibitory activity (50% inhibitory concentration, IC50) against recombinant Tv20S and purified human c20S, using fluorescent reporter substrates selective for each of the respective three catalytic subunits (β1, β2, and β5). Data are the mean and SD of the results of three independent experiments. Selectivity was calculated for the β5 subunit as the ratio of IC50 in human c20S over IC50 in Tv20S. Entries are listed in order of their β5 selectivity. CBP, carmaphycin B; MZB, marizomib; BTZ, bortezomib; ND, not determined.

To understand the mechanistic basis of parasite selectivity, we tested the same set of proteasome inhibitors against the purified human c20S proteasome, using peptide reporter substrates optimized for each of the three human catalytic subunits. Similar to *T. vaginalis*, all inhibitors inactivated the c20S β5 subunit, with IC50 values ranging from 0.16 to 4,102 nM, with the single exception of compound **75,** which had no measurable c20S β5 inhibitory activity but was also the least potent for Tv20S β5 inhibition (Table 1). Contrary to the findings with Tv20S, the majority of compounds also inactivated the human c20S β2 subunit, but generally at higher IC50 values compared to c20S β5 (Table 1). The human β1 subunit was only inactivated by the broadly active inhibitors, bortezomib, carmaphycin B, and marizomib, and only one of the *T. vaginalis*-selective inhibitors (**4** at a very high concentration). Also, as for Tv20S, the alkene derivative without epoxide (**104**) had no inhibitory activity against any of the human catalytic β subunits (Table 1).

Based on the IC50 data for the β5 subunits of *T. vaginalis* and human c20S, we calculated SI values for the β5 target activity and compared them with the SI values for whole-cell activity. Overall, a modest positive, albeit not quite significant, correlation was observed between the two activities (Fig. 5B). Importantly, for several inhibitors, including **8** and **12**, the selectivity for whole-cell activity closely matched the selectivity for β5 inhibition *in vitro* (Fig. 5A and B). In contrast, a non-selective inhibitor, **19**, showed similar β5 inhibition *in vitro* and GI50 values in whole cells for *T. vaginalis* and human cells (Fig. 5A and B). These data suggest that parasite selectivity of the most selective proteasome inhibitors can be explained by differential inhibition of the β5 subunit of the 20S proteasome. Furthermore, β5 inhibitory activity was positively and significantly correlated with whole-cell activity against *T. vaginalis*, with the best compounds displaying close to a 1:1 ratio, suggestive of proteasome inhibition as the major, if not only, determinant of anti-parasitic activity (Fig. 5C). Other compounds showed 10- to 100-fold lower whole-cell activity compared to β5 inhibition, which points to other factors (e.g., cellular uptake) beyond proteasome inhibition as being important for determining whole-cell activity.

### On-target activity of proteasome inhibitors in live *T. vaginalis*

**Fig 6:**
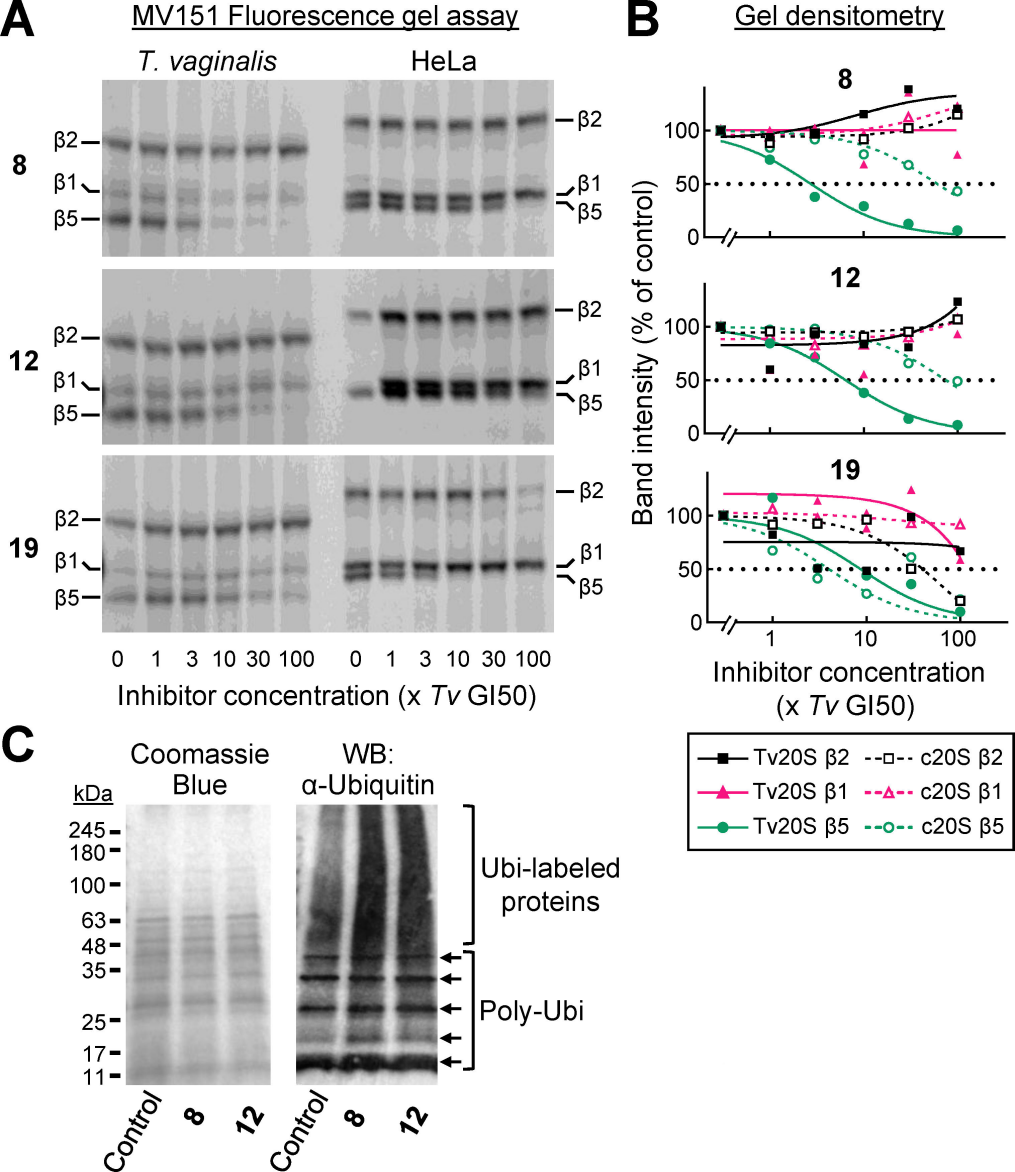
Differential proteasome subunit inactivation in live *T. vaginalis*. (**A**) *T. vaginalis* F1623 and human HeLa cells were incubated for 3 h with one of the three indicated proteasome inhibitors (bolded numbers, structures shown in Fig. 4) at concentrations of the indicated multiples of the respective GI50 values in *T. vaginalis*. Cell lysates were prepared, reacted with the fluorescent activity-based probe MV151, which binds to all three subunits of the eukaryotic proteasome, and analyzed by fluorescence gel electrophoresis. Catalytic subunit assignments were made based on their molecular weight, as confirmed by prior mass spectrometric studies (28). (**B**) Gel images were analyzed by densitometry, and individual band intensities were determined. Data points represent means of three independent experiments and are expressed relative to controls not incubated with inhibitors (with one exception for compound **12** in HeLa cells, where a loading error occurred in the controls without inhibitor in panel A, so the 1× *Tv* GI50 bands were used instead as controls). (**C**) Immunoblot analysis of ubiquitin-conjugated proteins in *T. vaginalis* lysates following 3 h treatment with a 30× GI50 concentration of the indicated proteasome inhibitors or a control (vehicle only, dimethyl sulfoxide) (right panel). Total protein was visualized by Coomassie brilliant blue staining (left panel). Arrows refer to the predicted poly-ubiquitins not attached to target proteins.

To confirm the subunit targeting in live cells, *T. vaginalis* and human HeLa cells were incubated with a range of concentrations of the two most selective proteasome inhibitors, **8** and **12**. Cell extracts were prepared and incubated with a fluorescent proteasome probe that labels the three catalytically active proteasome subunits (27, 30). Following protein separation on a denaturing gel, the three active subunits are visible as distinct bands in control cells (Fig. 6A). Consistent with the enzymatic assays, the gel assay showed that β5 was the primary target of both inhibitors in *T. vaginalis*, as revealed by the concentration-dependent loss of β5 labeling but only minimal changes in labeling of β1 and β2 (Fig. 6A). Quantitation of the gel bands by densitometry showed that β5 was inactivated in *T. vaginalis* at 22-fold and 12-fold lower concentrations by **8** and **12**, respectively, compared to human cells, while β1 and β2 labeling was not affected (Fig. 6B). For comparison, the non-selective proteasome inhibitor, **19**, inhibited β5 labeling at similar concentrations in live *T. vaginalis* and human cells (Fig. 6A and B), thereby also confirming the *in vitro* findings with purified 20S proteasomes. These results provide independent evidence of differential β5 inactivation as the primary mechanism of growth inhibition in *T. vaginalis*.

To further demonstrate the on-target activity of **8** and **12**, we treated live *T. vaginalis* with the inhibitors and assayed the abundance of ubiquitin-labeled proteins by immunoblotting using an antibody against ubiquitin. Both inhibitors led to an accumulation of ubiquitinated proteins with a broad range of molecular weights (Fig. 6C), consistent with the key role of the proteasome in the ubiquitin-initiated turnover of proteins in eukaryotic cells (15).

### Docking simulations of inhibitor selectivity for Tv20S

**Fig 7:**
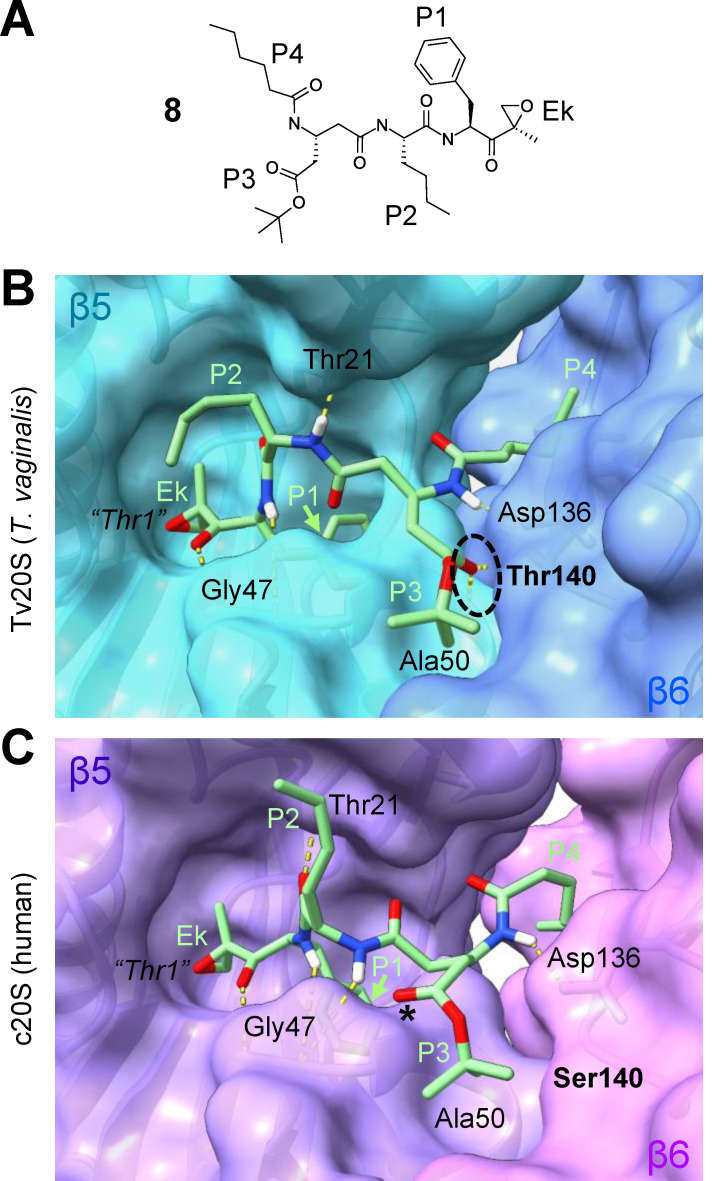
Docking simulations of inhibitor binding and selectivity for Tv20S. Non-covalent docking simulations were performed for the *T. vaginalis*-selective proteasome inhibitor **8**, depicted in (**A**), to the 3D structures of the β5 and β6 subunits of the 20S proteasome of *T. vaginalis* (Tv20S, **B**) and humans (c20S, **C**). To avoid spatial constraints and uncertainty about the exact linkage between inhibitor and target proteins, we considered the unreacted inhibitor with its epoxyketone (Ek) warhead intact and removed catalytic Thr1 in β5 (indicated in its approximate location as virtual *“Thr1”* in B and C) from both target proteins. The P1-P3 amino acid residues and the N-cap (P4) of the inhibitor are bolded in black in A and labeled in green in B and C. Predicted hydrogen bonds are represented with dashed yellow lines. The unique hydrogen bonds predicted in Tv20S β6 are circled, and the matching amino acid Thr140 is bolded (**B**). The corresponding amino acid in c20S (Ser140) is also bolded for comparison (**C**). The asterisk in C indicates the different predicted location of the carbonyl at P3 of the ligand in c20S compared to its location in Tv20S.

To begin to understand the structural basis of the selectivity of the parasite-selective β5 inhibitors, we performed docking calculations for the most selective compound, **8** (Fig. 7A), using the cryo-EM structures of Tv20S (27) and human c20S (31). Because the structure of the covalent bond that forms between the epoxyketone warhead of the inhibitor and the catalytic Thr1 of the β5 subunit is not known for **8**, we elected to minimize the modeling uncertainty by docking the complete, unreacted ligand and by removing the catalytic Thr1 of β5 for modeling to avoid steric collisions. Docking of **8** to the substrate-binding pocket along the β5 and β6 interface predicted similar poses for Tv20S and c20S with the epoxyketone group in the close proximity to the expected nucleophilic Thr1 of β5 for both proteins (depicted as virtual *“Thr1”* in Fig. 7B and C). These simulations suggest that the poses are consistent with the experimentally observed reactivity between ligand and target protein. Furthermore, the P1–P4 residues of the ligand were predicted to be generally accommodated in the equivalent respective pockets in the *T. vaginalis* and human target proteins (Fig. 7B and C). Furthermore, several potential hydrogen bonds were predicted between **8** and amino acid residues present in both Tv20S and c20S, namely between P1 and Gly47 of β5, P2 and Thr21 of β5, and P3 and Asp136 of β6 (Fig. 7B and C). Crucially, our modeling predicted two additional hydrogen bonds that formed only with the parasite but not the human proteasome. Thus, the carbonyl at P3 of **8** forms a hydrogen bond with the Thr140 of Tv20S β6 (circled in Fig. 7B), while the human β6 has a Ser140 in the same position that does not show the equivalent hydrogen bond (Fig. 7C). Furthermore, the same P3 carbonyl forms a second hydrogen bond with Ala50 in Tv20S β5, but the interaction does not occur with Ala50 in c20S β5, possibly due to Ser140 in c20S β6 repelling the carbonyl of the ligand so it assumes a different orientation (asterisk in Fig. 7C). To support these predictions, we modeled **94**, a derivative of **8** with a protected β-Glu residue at the P3 position. The model predicted that **94** would not form these critical hydrogen bond interactions with Thr140 or Ala50. This aligns well with our experimental results, which showed that **94** lacked activity and selectivity against *T. vaginalis* (see Fig. 2D). Together, these two unique additional hydrogen bonds predicted for the interactions of **8** with β5 and β6 residues of the *T. vaginalis* proteasome suggest a structural explanation of the high target selectivity of **8** for the parasite over human cells.

## DISCUSSION

This study expands on our prior findings that proteasome inhibition is a viable strategy for suppressing *T. vaginalis* growth and survival (22). Screening of a diverse library of proteasome inhibitors identified compounds with potencies against *T. vaginalis* in the 10–20 nM range and >200-fold selectivity over human cells. The current work also demonstrates that inhibition of the catalytic β5 subunit alone is sufficient to mediate the antiparasitic effects of proteasome inhibition, as all tested inhibitors targeted β5 with no notable inactivation of the β1 and β2 catalytic subunits. These findings indicate that Tv20S β5 has unique and critical functions in *T. vaginalis* that cannot be compensated for by β1 and β2.

While mammalian and microbial cells demonstrate a reliance on a functional β5 subunit for growth or viability (18, 32, 33), the underlying reasons are not clear. A possible explanation emerges from the nature of protein folding, with hydrophobic amino acids typically sequestered in a central core. For the proteasome to degrade these proteins, they must be unfolded to expose this hydrophobic interior (34). The β5 subunit, which exhibits chymotrypsin-like specificity for large hydrophobic residues (35), is thus strategically positioned to make critical initial cuts within these newly accessible core regions. Consequently, these initial cleavages by β5 may irreversibly destabilize protein substrates, thus serving as a gatekeeper for subsequent additional proteolytic steps that lead to complete protein breakdown into small peptides. Alternatively, it is conceivable that the critical functions of β5 may be explained by its ability to cleave targets that cannot be cleaved by the other catalytic subunits but are critical for the growth and survival of the parasite. Consistent with this notion, the β5 substrate preferences are not shared by β1 or β2 (28), although the specific protein targets of any of the catalytic subunits in live cells have not been identified.

The remarkable whole-cell selectivity of the top compounds for *T. vaginalis* over mammalian cells is closely matched by their *in vitro* target selectivity for the β5 proteasome subunit of the parasite, thus providing a sufficient mechanistic basis for their parasite selectivity. Interestingly, we also identified several inhibitors that were moderately selective in whole cells but showed minimal or no *in vitro* selectivity with purified proteasomes. These findings suggest that mechanisms other than molecular target selectivity can contribute to selectivity in whole cells. Although the specifics of such mechanisms are yet to be fully understood, they may involve differential inhibitor uptake or metabolism, or possibly additional targets in *T. vaginalis* compared to human cells (36). Defining these alternative mechanisms could pave the way for their systematic use in designing superior inhibitors with even greater parasite selectivity, potentially acting together with and extending beyond the benefits of β5-targeted approaches alone.

For the most promising compounds, their inhibitory potency *in vitro* against purified proteasomes closely matched their potency in whole cells. This suggests their anti-parasitic activity is primarily determined by their ability to inactivate the β5 subunit of the 20S proteasome within the cells. However, we also identified inhibitors whose whole-cell potency was 10- to 100-fold lower than their *in vitro* inhibitory activity for the purified proteasome. Although the underlying reasons are currently unknown, it is likely that those inhibitors most affected by activity loss in whole cells are compromised by instability, impaired cellular uptake, or extensive metabolism or export in the target cells (37, 38), thus limiting their effective intracellular concentration and ability to engage the target. Identification and consideration of such factors may be important in the further development of proteasome inhibitors as new agents against trichomoniasis.

The best proteasome inhibitors were active against several independently collected strains of *T. vaginalis*, underlining their potential therapeutic utility. Most importantly, they could overcome resistance to metronidazole, the drug most commonly used for the treatment of trichomoniasis (9). Resistance to this drug and other nitro-substituted heterocyclic antimicrobials occurs in up to 17% of cases in some studies (39), yet no FDA-approved alternatives presently exist, emphasizing the importance of developing treatment alternatives. The mechanism of metronidazole resistance involves increased export or diminished activation of the prodrug by reductases into the reactive forms of the drug (40). Our data show that these mechanisms do not impact the activity of proteasome inhibitors, presumably because the reductive pathway required for nitroheterocyclic drug activation is not important to the functioning of proteasome inhibitors.

Cytotoxicity testing showed that the leading proteasome inhibitors, while generally less active in mammalian cells than *T. vaginalis*, displayed variations in selectivity. In particular, the human B lymphoma cell line, Raji, of all tested mammalian cells, was most susceptible to two of the inhibitors that also had excellent potency in *T. vaginalis*. Raji cells, like all lymphocytes, also express an inducible form of the 20S proteasome (i20S) in which 22 of the 28 subunits are identical to c20S, but the catalytic subunits, β1, β2, and β5, are each replaced with different catalytic subunits, β1i, β2i, and β5i, respectively. The i20S is involved in specialized roles such as MHC class I antigen presentation, cytokine modulation, and T-cell differentiation, making it an attractive therapeutic target for autoimmune disorders. Since many compounds in our screen were initially designed as i20S inhibitors, it is not surprising that Raji B cells with their ~50% i20S content (41) were more susceptible to some of the proteasome inhibitors when compared to the non-immune cells, HeLa, 3T3, and Vero, which contain minimal or no i20S. These findings raise the possibility that differential targeting of Tv20S might be harder to achieve relative to i20S compared to c20S. However, even if so, this may not be a major drug development concern. Preclinical studies in mice and non-human primates showed that the i20S inhibitor zetomipzomib did not significantly affect T-dependent antibody responses or the number of circulating lymphocytes at therapeutic concentrations (24, 42), which suggests a more targeted immunomodulatory effect rather than broad immunosuppression. These observations have implications for the future development of Tv20S inhibitors, as they suggest that such compounds will likely avoid targeting host c20S and may partially inhibit i20S in immune cells but without causing significant immune suppression, particularly if treatment of trichomoniasis is transient and short term.

Docking simulations of the most selective proteasome inhibitor (8) with Tv20S and c20S predicted two additional hydrogen bonds between the P3 side chain of the inhibitor and the β5 and β6 subunits of Tv20S. These specific interactions are not predicted to occur with human c20S due to residue substitution at the corresponding β6 site. Hydrogen bonds generally contribute around 1–2 kcal/mol to the binding energy of a ligand to its target, which would be consistent with the observed ~100-fold selectivity of the inhibitor for *T. vaginalis* over human cells (43). While the overall structures of Tv20S and c20S are very similar, subtle amino acid differences in the substrate-binding pockets are likely to be critical for differential inhibitor binding and activity. If such differences directly affect ligand binding, as predicted in our case, it can provide a compelling mechanism of differential drug action. However, other scenarios are conceivable where amino acid differences do not directly impact ligand binding but rather modify critical pockets or binding locations (27). Thus, it appears likely that more than one structural mechanism can be responsible for selectivity, perhaps involving different parts of the ligand. This raises the intriguing possibility that different mechanisms may be exploited in synergistic fashion by combining unique features of the ligands for future improvement of potency and selectivity toward realizing proteasome inhibition as a novel therapeutic strategy for trichomoniasis.

Preliminary predictions of a range of absorption, distribution, metabolism and excretion (ADME) properties of the two best proteasome inhibitors identified in our studies suggest that they have many features potentially compatible with both topical and oral administration, including acceptable aqueous solubility, permeability across epithelia, and oral absorption (Table S2). However, a major challenge with all peptide drugs is their susceptibility to proteases in the host, often leading to very short half-lives after oral or systemic dosing (44). Although stability and distribution studies have not yet been done for our lead compounds, further development of proteasome inhibitors for the treatment of trichomoniasis will need to consider these pharmacokinetic challenges and potentially make modifications of the inhibitors, such as peptoid backbones or macrocyclization, that have been shown to improve stability in other systems (45, 46) and test their impact on drug stability and tissue levels in suitable *in vitro* and *in vivo* models.

## MATERIALS AND METHODS

### Library of proteasome inhibitors

A library of 373 proteasome inhibitors was assembled from several academic and commercial sources (19, 28, 41, 47–49). Bortezomib, Ixazomib, Marizomib, Carfilzomib, Delanzomib, Epoxomicin, and Oprozomib were purchased from Medchemexpress. Most of the inhibitors were N-capped tripeptides with different combinations of natural and non-natural amino acids and different C-terminal groups designed to inactivate the catalytic sites of the target proteasomes (Table S1). Stocks of the test agents were dissolved in dimethyl sulfoxide (DMSO) and stored at −20°C.

### Synthesis of new proteasome inhibitors

Several new proteasome inhibitors were synthesized to test specific hypotheses about SARs. The syntheses followed established chemical protocols (47), incorporating slight modifications to optimize yields and reproducibility. Broadly, the central core dipeptide (P2 and P3) was first assembled via established peptide chemistry and subsequently connected to the N-cap moiety. The sensitive epoxy ketone (P1 fragment) was then, in the last step, connected to the main fragment (see scheme in Fig. 3B). Specifically, synthesis of epoxyketone (P1) was accomplished using the enone obtained from L-Phe or L-biphenyl alanine. Specifically, the epoxide was introduced by oxidizing the Michael acceptor with regular household bleach (Chlorox; Chlorox Co.) where the two diastereomers were obtained in a 5:1 ratio in favor of the *R*-diastereomer, which was separated from the minor *S*-diastereomer by silica flash-chromatography using 50% diethyl ether in hexane as the mobile phase. To create the peptide bond between the epoxy ketone (P1) and P2, the *tert*-butyl group was next converted to the corresponding carboxylic acid using TFA in DCM for exactly 45 min at room temperature. After removal of the Boc group attached to P1 and subsequent peptide coupling with P2, the target compounds were formed. Compounds were purified by flash chromatography. The identity of the compounds was confirmed by ^1^H NMR and high-resolution mass spectrometry, and purity was estimated to be >95%.

### Antimicrobial and cytotoxicity assays

The following *T. vaginalis* strains were used: F1623 (50), G3 (ATCC PRA-98), and LA1 (25). Parasites were grown at 37°C in TYM Diamond’s medium supplemented with 180 µM ferrous ammonium sulfate (51). Drug susceptibility assays were performed as described previously (23) with several minor modifications. Briefly, 10 mM stocks of the test compounds in DMSO were serially diluted 1:3 using an Echo 650 Acoustic Liquid Handler (Beckman Coulter). Subsequently, trophozoites (5 × 10^3^/well) in TYM modified media were added to the wells in 384-well plates, and cultures were incubated for 24 h at 37°C under anaerobic conditions (AnaeroPack-Anaero System; Remel). For testing in mammalian cells, the following cell lines were used: Human cervical cancer cells HeLa (ATCC CCL-2), non-transformed mouse fibroblast cell line 3T3-L1 (ATCC CL-173), normal monkey kidney epithelial cell line Vero (ATCC CCL-81), and human B lymphoma cells Raji (ATCC CCL-86). Test compounds were serially diluted (1:3) and added to mammalian cell cultures grown in 384-well plates, and cultures were incubated for 48 h at 37°C in 5% CO_2_, 95% air. Growth and viability of *T. vaginalis* and mammalian cells were determined with an ATP assay by adding the BacTiter-Glo Microbial Cell Viability Assay reagent (Promega) directly to the wells and measuring ATP-dependent luminescence in a microplate reader. The 50% growth inhibition concentration (GI50) was derived from the concentration-response curves using GraphPad Prism (GraphPad Software). Experiments were repeated at least three times, and the mean and SEM of the pGI50 values were calculated.

### *In vitro* proteasome assays

The *T. vaginalis* proteasome was expressed in recombinant form as previously described (27). Briefly, *Spodoptera frugiperda* cells were co-infected with three baculovirus vectors carrying the *T. vaginalis* genes for the 7 α-subunits, 7 β-subunits, and the Ump-1 chaperone, respectively. The resulting proteasome complex was purified from cell lysates via streptavidin column and size exclusion chromatography and stored at −80°C. Purified human c20S proteasome and recombinant human proteasome activator PA28α (21) were obtained from Bio-Techne and stored at −80°C. Dilutions of the test agents were transferred to 384-well plates (Greiner Bio-One) in triplicate wells using an Echo 650 Acoustic Liquid Handler (Beckman Coulter). Six replicate plates were prepared for each compound for testing with three *T. vaginalis*-optimized substrates and three human-optimized substrates. After dispensing, plates were sealed and stored at −20°C. The following fluorescent substrates and final substrate concentrations were used to assay subunit activities of the *T. vaginalis* proteasome (Tv20S) (28) or the human constitutive proteasome (c20S) (19, 52): Ac-RYFD-amc (Genscript) at 100 µM for Tv20S β1 subunit, Ac-FRSR-amc (Genscript) at 20 µM for Tv20S β2 subunit, Ac-GWYL-amc (Genscript) at 20 µM for Tv20S β5 subunit, z-LLE-amc (R&D Systems) at 80 µM for c20S β1 subunit, z-VLR-amc (AdipoGen Life Sciences) at 30 µM for c20S β2 subunit, and suc-LLVY-amc (R&D Systems) at 65 µM for c20S β5 subunit. Substrates and proteasomes were diluted in an assay buffer consisting of HEPES pH 7.5 and 1 mM DTT. For the human proteasome assays, 2 nM c20S was incubated at room temperature with 200 nM PA28α for 1 h to activate the complex prior to addition to the 384-well plates containing the substrate-inhibitor mixtures. The recombinant Tv20S did not require activation. Both proteasomes were added to the wells at a 1 nM final assay concentration. Fluorescence was measured over 4 h at 360 nm excitation and 460 nm emission on a Biotek Synergy HTX plate reader. A Vmax was calculated from eight kinetic reads from each well between 30 and 105 min, and the data were analyzed by 4-parameter logistic dose-response curves using Graphpad Prism 10.2.2 to derive 50% inhibitory concentrations (IC50).

### Fluorescence gel assays of proteasome subunit targeting

*T. vaginalis* F1623 and human HeLa cells were incubated for 3 h with proteasome inhibitors at a range of concentrations or with solvent as a control. Cells were lysed by three consecutive freeze/thaw cycles in a buffer of 50 mM HEPES, pH 7.5, 10 µM E64, 100 µM AEBSF, 10 µM RR11a, 1 µM Pepstatin, and 1 mM DTT. Protein concentrations were measured with the Pierce BCA assay (Thermo Scientific) and adjusted to 5 mg/mL. Samples were incubated with 2 µM Me4BodipyFL-Ahx3Leu3VS (53) at 2.5 mg/mL protein concentration for 24 h at 37°C. Following this incubation, samples were separated on 12% Bis-Tris gels (Thermo) in MOPS SDS buffer (Invitrogen) at 130 V. The gels were imaged on a Bio-Rad ChemiDoc XRS + at 470  nm, and excitation 530 nm emission. Band intensities were quantified using ImageJ (NIH). Each peak corresponding to a protein band was selected, and the area under the curve was determined after subtraction of the intensity in an adjacent area outside of the respective band. For closely spaced bands, the midpoint between the peaks of two bands was used to separate the areas. Band intensities were normalized to the respective controls, not incubated with inhibitors.

### Immunoblot analysis of ubiquitin labeling 

*T. vaginalis* F1623 were incubated for 3 h with 30× GI50 of the tested proteasome inhibitor or solvent control, and cells were lysed using RIPA buffer with added Halt protease inhibitors (Thermo Scientific). Protein concentrations were measured using the Pierce BCA assay (Thermo Scientific) and adjusted to 10 mg/mL. Proteins were fractionated on SDS-PAGE, transferred onto a nitrocellulose membrane (Bio-Rad), which was blocked with 5% non-fat dry milk in TBST buffer (20 mM Tris base, 150 mM NaCl, 0.05% Tween 20, pH adjusted to 7.6 with HCl) for 1 h and probed with primary anti-ubiquitin antibody (Proteintech, 10201-2-AP) at a 1:2,000 dilution, followed by incubation with anti-rabbit horseradish peroxidase (HRP)-conjugated IgG (Jackson laboratory) at a 1:5,000 dilution. HRP labeling was visualized by chemiluminescence (Immobilon, Millipore).

### Molecular modeling, docking simulations, and predictions of ADME

The 3D structure of Tv20S, complexed with the inhibitor CP-17, was obtained from the Protein Data Bank (PDB ID 8P0T) (27). The structure of the human proteasome c20S, complexed with bortezomib, was used for comparison (PDB 5LF3) (54). MGLTools (55) was used to prepare the target proteins for docking. We only considered the β5 and β6 subunits since a prior study had shown that those subunits are critical for the binding of proteasome inhibitors (56, 57). Furthermore, all non-standard residues (including water and ions) were deleted. In addition, we deleted the catalytic Thr1 residue from the β5 subunit to better reproduce the experimentally confirmed binding pose of a known ligand (CP-17) to Tv20S (27) compared to the protein with Thr1 when using non-covalent docking. We chose non-covalent docking for modeling, so we could examine the interaction of ligands with the target proteins just prior to the occurrence of the reaction that leads to covalent binding. A grid box of 10 Å was defined around the site of the complexed ligand for docking purposes. The 3D structure of the ligand was prepared using OpenBabel (58) at pH 7.4 with Gasteiger partial charges. AutoDock-GPU (59) was used to perform non-flexible docking. The docking protocol was validated by re-docking CP-17 to Tv20S and found to closely mimic the experimentally determined binding pose (27), including the location of the expected covalent site. Docking simulations considered 10 generated poses, from which the best pose was chosen by visual inspection (60) based on the correct location of the reactive site of the ligand compared to the experimentally confirmed structure (27). The results from docking were visualized with ChimeraX (61). Hydrogen bonding interactions were identified using the built-in hbonds tool. ADME predictions were generated using Schrödinger QikProp (release 2025-3), starting from compound conformations generated by ligprep.

## SUPPLEMENTAL MATERIAL

Supplemental material aac.00893-25-s0001.pdf Tables S1 and S2.

ASM does not own the copyrights to Supplemental Material that may be linked to, or accessed through, an article. The authors have granted ASM a non-exclusive, world-wide license to publish the Supplemental Material files. Please contact the corresponding author directly for reuse.
