# Supplementary material for: Inhibitors of the 20S proteasome β5 subunit as potent and selective agents against Trichomonas vaginalis
Source: Antimicrob Agents Chemother. 2025 Nov 11;69(12):e00893-25. doi: 10.1128/aac.00893-25 (PMC12691669; doi:10.1128/aac.00893-25)
Supplement: Supplemental material — Tables S1 and S2. [file aac.00893-25-s0001.pdf]

Supplemental Materials

Inhibitors of the 20S proteasome β5 subunit as potent and selective agents against *Trichomonas vaginalis*

Yukiko Miyamoto, Brianna M. Hurysz, Pavla Fajtova, Peter Eckmann,

Mateus Sá Magalhães Serafim, Thales Kronenberger, Momen Al-Hindy, Patrick M. Dekker,

Elmer Maurits, Herman S. Overkleeft, B. Mikael Bergdahl, Jehad Almaliti,

William H. Gerwick, Anthony J. O'Donoghue and Lars Eckmann

Supplemental Table 1. Activities of proteasome inhibitors against *Trichomonas vaginalis* and human cells

Supplemental Table 2. Predicted ADME properties of top proteasome inhibitors

Supplemental Table 1

Compound ID	*T. vaginalis* F1623 pGI50 (mean ± SE)	*T. vaginalis* F1623 GI50 (µM)	HeLa pGI50 (mean ± SE)	HeLa GI50 (µM)	Selectivity Index	SMILES
I. Active against *T. vaginalis*						
**1**	7.95 ± 0.12	0.01	7.36 ± 0.15	0.04	3.89	O=C([C@@]1(OC1)C)[C@H](CC2=CC=C(F)C=C2F)NC([C@H](CCCC)NC([C@H](CC(N3CCCC3)=O)NC([C@@H](N4CCCC4=O)CC5=CC=C(OC)C=C5)=O)=O)=O
**2**	7.89 ± 0.25	0.01	7.19 ± 0.19	0.06	5.01	O=C([C@@]1(OC1)C)[C@H](CC2=CC=C(F)C=C2F)NC([C@H](CC3=CC=C(F)C=C3)NC([C@H](CC(N4CCCC4)=O)NC([C@@H](N5CCCC5=O)CC6=CC=C(OC)C=C6)=O)=O)=O
**3**	7.72 ± 0.14	0.02	6.16 ± 0.08	0.69	36.31	COC1=CC=C(C[C@H](NC(=O)[C@H](C)NC(=O)CN2CCOCC2)C(=O)N[C@@H](CC2=CC=C(C=C2)C2=CC=CC=C2)C(=O)[C@@]2(C)CO2)C=C1
**4**	7.71 ± 0.18	0.02	6.38 ± 0.09	0.41	21.38	COC1=CC=C(C[C@H](NC(=O)[C@H](C)NC(=O)C2=C(C)C3=C(C2)C=CC=C3)C(=O)N[C@@H](CC2=CC=CC=C2)C(=O)[C@@]2(C)CO2)C=C1
**5**	7.67 ± 0.30	0.02	6.58 ± 0.09	0.26	12.30	CCCCCC(=O)N[C@@H](CC1=CC=C(O)C=C1)C(=O)N[C@@H](CCCC)C(=O)N[C@@H](CC1=CC=CC=C1)C(=O)[C@@]1(C)CO1
**6**	7.63 ± 0.10	0.02	7.05 ± 0.11	0.09	3.80	COc1ccc(C[C@H](NC(=O)[C@H](Cc2cccc3ccccc23)NC(=O)CN2CCOCC2)C(=O)N[C@@H](CC(C)C)C(=O)[C@@]2(C)CO2)cc1
**7**	7.51 ± 0.03	0.03	6.98 ± 0.04	0.10	3.39	COC1CCC(C[C@H](NC(=O)[C@@H](Cc2ccccc2)N=[N+]=[N-])C(=O)N[C@@H](Cc2ccccc2)C(=O)N[C@@H](CC(C)C)C(=O)[C@@]2(C)CO2)CC1
**8**	7.45 ± 0.19	0.04	5.11 ± 0.05	7.78	218.78	O=C(N[C@H](C(N[C@@H](CC1=CC=CC=C1)C([C@@]2(C)CO2)=O)=O)CCCC)C[C@H](CC(OC(C)(C)C)=O)NC(CCCCC)=O
**9**	7.35 ± 0.07	0.04	7.14 ± 0.06	0.07	1.62	CC(C)C[C@H](NC(=O)[C@H](C)NC(=O)[C@H](CC12CC3CC(CC(C3)C1)C2)N=[N+]=[N-])C(=O)N[C@@H](CC(C)C)C(=O)[C@@]1(C)CO1
**10**	7.30 ± 0.05	0.05	6.51 ± 0.09	0.31	6.17	CC(C)C[C@H](NC(=O)[C@H](Cc1ccccc1)NC(=O)[C@H](Cc1cccc2ccccc12)NC(=O)[C@H](Cc1ccccc1)N=[N+]=[N-])C(=O)[C@@]1(C)CO1
**11**	7.17 ± 0.23	0.07	5.46 ± 0.11	3.46	51.29	COC1=CC=C(C[C@H](NC(=O)[C@H](C)NC(=O)OCC2=CC=CC=C2)C(=O)N[C@@H](CC2=CC=CC=C2)C(=O)[C@@]2(C)CO2)C=C1
**12**	7.16 ± 0.25	0.07	5.36 ± 0.13	4.33	63.10	COC1=CC=C(C[C@H](NC(=O)[C@@H](C)NC(=O)OCC2=CC=CC=C2)C(=O)N[C@@H](CC2=CC=CC=C2)C(=O)[C@@]2(C)CO2)C=C1

**13**	7.07 ± 0.24	0.08	7.08 ± 0.07	0.08	0.98	CC[C@H](C)[C@H](NC(=O)CCCCC#C)C(=O)N[C@@H]([C@@H](C)CC)C(=O)N[C@@H]([C@@H](C)O)C(=O)N[C@@H](CC(C)C)C(=O)[C@@]1(C)CO1
**14**	7.00 ± 0.28	0.10	5.61 ± 0.29	2.47	24.55	O=C([C@H](CC1=C(C(F)=C(C(F)=C1F)S(C2=CC=CC(C3)=C2)(=O)=O)F)NC([C@@H](NC3=O)C(C)C)=O)N[C@H](C([C@@]4(OC4)C)=O)CC(C)C
**15**	7.00 ± 0.73	0.10	8.91 ± 0.20	0.001	0.01	C[C@@]1(CO1)C([C@H](CC(C)C)NC([C@H](CS(CCCCCCCCC2)(=O)=O)NC([C@H](C(C)C)NC2=O)=O)=O)=O
**16**	6.99 ± 0.01	0.10	6.40 ± 0.07	0.40	3.89	CC(C)C[C@H](NC(=O)[C@@H](NC(=O)N[C@@H](C(C)C)C(=O)NCc1ccccc1)C(C)C)C(=O)N[C@@H](CC(C)C)C(=O)[C@@]1(C)CO1
**17**	6.97 ± 0.06	0.11	6.98 ± 0.04	0.11	0.98	CC(C)C[C@H](NC(=O)[C@H](Cc1ccccc1)NC(=O)[C@H](Cc1c[nH]c2ccccc12)NC(=O)[C@H](Cc1ccccc1)N=[N+]=[N-])C(=O)[C@@]1(C)CO1
**18** (Marizomib)	6.91 ± 0.46	0.12	6.19 ± 0.16	0.65	5.25	C[C@]12[C@H](C(=O)N[C@]1(C(=O)O2)[C@H]([C@H]3CCCC=C3)O)CCCl
**19**	6.91 ± 0.23	0.12	6.90 ± 0.08	0.13	1.02	CCCCCC(=O)N[C@@H](CO)C(=O)N[C@@H](CCCC)C(=O)N[C@@H](CC1=CC=CC=C1)C(=O)[C@@]1(C)CO1
**20**	6.88 ± 0.17	0.13	5.67 ± 0.08	2.13	16.22	COC1=CC=C(C[C@H](NC(=O)[C@H](C)NC(=O)C2=C(C)C3=C(C2)C=CC=C3)C(=O)N[C@@H](CC2=CC=C(C=C2)C2=CC=CC=C2)C(=O)[C@@]2(C)CO2)C=C1
**21**	6.87 ± 0.07	0.14	6.31 ± 0.04	0.49	3.63	CC(C)C[C@H](NC(=O)[C@H](Cc1ccccc1)NC(=O)[C@@H](NC(=O)[C@@H](Cc1ccccc1)N=[N+]=[N-])C1CCCCC1)C(=O)[C@@]1(C)CO1
**22**	6.86 ± 0.24	0.14	5.64 ± 0.16	2.28	16.60	COC1=CC=C(C[C@H](NC(=O)[C@H](C)NC(=O)C2=C(C)C3=C(C2)C=CC=C3)C(=O)N[C@@H](CC2=CC=CC=C2)C(=O)[C@]2(C)CO2)C=C1
**23**	6.83 ± 0.00	0.15	5.90 ± 0.30	1.27	8.51	CC(C)C[C@H](NC(=O)[C@H](Cc1ccccc1)NC(=O)[C@H](CC1CCCCC1)NC(=O)[C@H](Cc1ccccc1)N=[N+]=[N-])C(=O)[C@@]1(C)CO1
**24**	6.82 ± 0.08	0.15	5.88 ± 0.07	1.32	8.71	CC(C)C[C@H](NC(=O)[C@H](Cc1ccccc1)NC(=O)[C@H](CC1CC2CCC1C2)NC(=O)[C@@H](Cc1ccccc1)N=[N+]=[N-])C(=O)[C@@]1(C)CO1
**25**	6.77 ± 0.06	0.17	6.24 ± 0.04	0.58	3.39	CC(C)C[C@H](NC(=O)[C@H](Cc1ccccc1)NC(=O)[C@H](CCC1CCCCC1)NC(=O)[C@@H](Cc1ccccc1)N=[N+]=[N-])C(=O)[C@@]1(C)CO1
**26**	6.70 ± 0.21	0.20	5.30 ± 0.08	5.03	25.12	COc1ccc(C[C@H](NC(=O)[C@H](C)NC(=O)C2=C(C)c3ccccc3C2)C(=O)N[C@@H](CC2CCCCC2)C(=O)[C@@]2(C)CO2)cc1
**27**	6.70 ± 0.07	0.20	6.16 ± 0.03	0.69	3.47	CC(C)C[C@H](NC(=O)[C@H](Cc1ccccc1)NC(=O)[C@@H](NC(=O)[C@@H](Cc1ccccc1)N=[N+]=[N-])c1ccccc1)C(=O)[C@@]1(C)CO1

**28**	6.70 ± 0.03	0.20	7.11 ± 0.03	0.08	0.39	CC(C)C[C@H](NC(=O)C(CC(C)C)NC(=O)[C@H](CC(C)C)NC(=O)OCc1ccccc1)B1OC2[C@@H]3C[C@H](C[C@]2(C)O1)C3(C)C
**29**	6.69 ± 0.03	0.21	6.11 ± 0.08	0.77	3.80	CC(C)C[C@H](NC(=O)[C@H](Cc1ccccc1)NC(=O)[C@H](CC1CCC(C)CC1)NC(=O)[C@@H](Cc1ccccc1)N=[N+]=[N-])C(=O)[C@@]1(C)CO1
**30**	6.68 ± 0.12	0.21	7.06 ± 0.07	0.09	0.42	CC(C)C[C@H](NC(=O)[C@@H](NC(=O)N[C@@H](C(C)C)C(=O)OC(C)(C)C)C(C)C)C(=O)N[C@@H](CC(C)C)C(=O)[C@@]1(C)CO1
**31**	6.68 ± 0.62	0.21	7.12 ± 0.18	0.08	0.36	O=C([C@]1(C)OC1)[C@@H](NC([C@@H](NC([C@@H](NC(CCCCC)=O)C(C)C)=O)CC2=CNC3=C2C=CC=C3)=O)CC4=CC=CC=C4
**32**	6.67 ± 0.09	0.21	6.66 ± 0.01	0.22	1.02	CC(C)C[C@H](NC(=O)[C@H](CC12CC3CC(CC(C3)C1)C2)NC(=O)c1cnccn1)B1OC2C[C@@H]3C[C@@H](C3(C)C)[C@]2(C)O1
**33**	6.61 ± 0.05	0.25	7.02 ± 0.11	0.10	0.39	CCC(C)C(NC(=O)CN=[N+]=[N-])C(=O)NC(C(C)CC)C(=O)NC(C(C)O)C(=O)NC(CC(C)C)C(=O)C1(C)CO1
**34**	6.60 ± 0.20	0.25	5.61 ± 0.24	2.44	9.77	C[C@H](NC(CCCCC)=O)C(NC(C(NC(C([C@@]1(C)CO1)=O)CC2=CC=C(F)C=C2F)=O)Cc3c(F)c(F)c(F)c(F)c3F)=O
**35**	6.60 ± 0.12	0.25	6.06 ± 0.06	0.88	3.47	OC(=O)C(F)(F)F.COc1ccc(C[C@H](NC(=O)[C@H](C)NC(=O)CN2CCOCC2)C(=O)N[C@@H](Cc2ccc3ccccc3c2)C(=O)[C@@]2(C)CO2)cc1
**36**	6.57 ± 0.10	0.27	6.45 ± 0.08	0.35	1.32	CC(C)C[C@H](NC(=O)[C@@H](NC(=O)N[C@@H](C(C)C)C(=O)NCc1ccccc1)C(C)C)C(=O)N[C@@H](Cc1ccccc1)C(=O)[C@@]1(C)CO1
**37**	6.57 ± 0.10	0.27	6.56 ± 0.05	0.27	1.02	CC(C)C[C@H](NC(=O)[C@H](Cc1ccccc1)NC(=O)[C@H](CCc1ccccc1)NC(=O)[C@@H](Cc1ccccc1)N=[N+]=[N-])C(=O)[C@@]1(C)CO1
**38** (Carmaphycin	6.52 ± 0.12	0.30	8.71 ± 0.11	0.00	0.01	CCCCCC(=O)N[C@@H](C(C)C)C(=O)N[C@@H](CCS(=O)(=O)C)C(=O)N[C@@H](CC(C)C)C(=O)[C@]1(CO1)CCCCCCC(=O)N[C@@H](C(C)C)C(=O)N[C@@H](CCS(=O)(=O)C)C(=O)N[C@@H](CC(C)C)C(=O)[C@]1(CO1)C
**39**	6.50 ± 0.45	0.32	5.85 ± 0.06	1.43	4.47	C[C@@]1(C([C@@H](NC([C@H](CC2=CC=C(F)C=C2)NC([C@@H](NC(CCCCC)=O)CC(N(CC)CC)=O)=O)=O)CC3=C(F)C=C(F)C=C3)=O)OC1
**40** (Oprozomib)	6.47 ± 0.00	0.34	7.05 ± 0.04	0.09	0.26	COC[C@H](NC(=O)[C@H](COC)NC(=O)c1cnc(C)s1)C(=O)N[C@@H](Cc1ccccc1)C(=O)C1(C)CO1
**41**	6.46 ± 0.76	0.35	7.01 ± 0.38	0.10	0.28	O=C([C@]1(C)OC1)[C@@H](NC([C@@H](NC([C@@H](NC(CCCCC)=O)CC2=CNC3=C2C=CC=C3)=O)CC4=CC=CC5=C4C=CC=C5)=O)CC6=CC=CC=C6

**42** (Ixazomib)	6.44 ± 0.14	0.36	6.58 ± 0.09	0.26	0.72	B([C@H](CC(C)C)NC(=O)CNC(=O)C1=C(C=CC(=C1)Cl)Cl)(O)O
**43** (Bortezomib)	6.43 ± 0.10	0.38	7.50 ± 0.13	0.03	0.09	B([C@H](CC(C)C)NC(=O)[C@H](CC1=CC=CC=C1)NC(=O)C2=NC=CN=C2)(O)O
**44**	6.41 ± 0.09	0.38	6.77 ± 0.02	0.17	0.44	COc1ccc(C[C@H](NC(=O)[C@H](Cc2c[nH]c3ccccc23)NC(=O)CN2CCOCC2)C(=O)N[C@@H](CC(C)C)C(=O)[C@@]2(C)CO2)cc1
**45**	6.40 ± 0.09	0.40	5.93 ± 0.11	1.17	2.95	O=C([C@@]1(OC1)C)[C@H](CC2=CC=C(F)C=C2F)NC([C@H](CCCC)NC([C@@H](CC(N3CCCC3)=O)NC([C@@H](N4CCCC4=O)CC5=CC=C(OC)C=C5)=O)=O)=O
**46**	6.39 ± 0.13	0.41	5.68 ± 0.04	2.08	5.13	COc1ccc(C[C@H](NC(=O)[C@H](C)NC(=O)[C@H](Cc2ccccc2)N=[N+]=[N-])C(=O)N[C@@H](CC2CCCCC2)C(=O)[C@@]2(C)CO2)cc1
**47**	6.39 ± 0.09	0.40	6.60 ± 0.04	0.25	0.62	CC(C)C[C@H](NC(=O)[C@H](Cc1ccccc1)NC(=O)[C@H](Cc1ccc2ccccc2c1)NC(=O)[C@H](Cc1ccccc1)N=[N+]=[N-])C(=O)[C@@]1(C)CO1
**48**	6.33 ± 0.05	0.47	6.92 ± 0.05	0.12	0.26	CC[C@H](C)[C@H](NC(C)=O)C(=O)N[C@@H]([C@@H](C)CC)C(=O)N[C@@H]([C@@H](C)O)C(=O)N[C@@H](CC(C)C)C(=O)[C@@]1(C)CO1
**49** (Epoxomicin)	6.33 ± 0.05	0.47	7.64 ± 0.30	0.02	0.05	CC[C@H](C)[C@@H](C(=O)N[C@@H]([C@@H](C)O)C(=O)N[C@@H](CC(C)C)C(=O)[C@]1(CO1)C)NC(=O)[C@H]([C@@H](C)CC)N(C)C(=O)C
**50**	6.31 ± 0.37	0.49	7.87 ± 0.43	0.01	0.03	CC(C)C(C(NC(CC1=C(F)C(F)=C(F)C(F)=C1F)C(N[C@@H](CC(C)C)C([C@]2(C)OC2)=O)=O)=O)NC(CC3=CC=C(SC)C=C3)=O
**51**	6.30 ± 0.44	0.50	7.52 ± 0.22	0.03	0.06	O=C(N[C@@H](CC1=CC=CC=C1)C(N[C@@H](CCS(CCN)(=O)=O)C(N[C@@H](CC2=CC=CC=C2)C([C@]3(CO3)C)=O)=O)=O)CC4=CC=CC=C4
**52**	6.29 ± 0.51	0.51	7.37 ± 0.05	0.04	0.08	O=C([C@]1(C)OC1)[C@@H](NC([C@@H](NC([C@@H](NC(CCCCC)=O)CC2=C(C=CC=C3)C3=CC=C2)=O)CC4=CNC5=C4C=CC=C5)=O)CC6=CC=CC=C6
**53**	6.28 ± 0.08	0.52	6.77 ± 0.20	0.17	0.32	COc1ccc(C[C@H](NC(=O)[C@@H](Cc2ccccc2)N=[N+]=[N-])C(=O)N[C@@H](Cc2ccccc2)C(=O)N[C@@H](CC(C)C)C(=O)[C@@]2(C)CO2)cc1
**54**	6.27 ± 0.09	0.54	6.80 ± 0.08	0.16	0.30	CCC(C)[C@H](NC(=O)[C@H](C(C)CC)N(CC=C)C(C)=O)C(=O)N[C@@H](C(C)O)C(=O)N[C@@H](CC(C)C)C(=O)[C@@]1(C)CO1
**55**	6.24 ± 0.10	0.58	6.98 ± 0.04	0.11	0.18	CC(C)C[C@H](NC(=O)[C@H](Cc1ccccc1)N=[N+]=[N-])C(=O)N[C@@H](CC12CC3CC(CC(C3)C1)C2)C(=O)N[C@@H](CC(C)C)C(=O)[C@@]1(C)CO1
**56**	6.24 ± 0.15	0.57	7.30 ± 0.04	0.05	0.09	CC(C)C[C@H](NC(=O)OCc1ccccc1)C(=O)N[C@@H](CC12CC3CC(CC(C3)C1)C2)C(=O)N[C@@H](CC(C)C)C(=O)[C@@]1(C)CO1

**57**	6.22 ± 0.03	0.61	5.80 ± 0.05	1.57	2.63	CC(C)C[C@H](NC(=O)[C@H](Cc1ccccc1)NC(=O)[C@H](CC1CCC2CCCCC2C1)NC(=O)[C@@H](Cc1ccccc1)N=[N+]=[N-])C(=O)[C@@]1(C)CO1
**58**	6.22 ± 0.14	0.60	6.42 ± 0.10	0.38	0.63	COc1ccc(C[C@H](NC(=O)[C@H](Cc2ccc3ccccc3c2)NC(=O)CN2CCOCC2)C(=O)N[C@@H](CC(C)C)C(=O)[C@@]2(C)CO2)cc1
**59**	6.21 ± 0.11	0.62	7.01 ± 0.07	0.10	0.16	CC(C)C[C@H](NC(=O)[C@H](Cc1ccccc1)NC(=O)c1cnccn1)B1OC2[C@@H]3C[C@H](C[C@]2(C)O1)C3(C)C
**60**	6.11 ± 0.19	0.77	5.20 ± 0.10	6.31	8.13	COC1=CC=C(C[C@H](NC(=O)[C@H](C)NC(=O)C2=C(C)C3=C(C2)C=CC=C3)C(=O)N[C@@H](CC2=CC=C(C=C2)C2=CC=CC=C2)C(=O)[C@]2(C)CO2)C=C1
**61**	6.10 ± 0.13	0.79	6.71 ± 0.03	0.20	0.25	CC(C)C[C@H](NC(=O)[C@H](Cc1c(F)c(F)c(F)c(F)c1F)NC(=O)[C@H](Cc1ccccc1)NC(=O)[C@H](Cc1ccccc1)N=[N+]=[N-])C(=O)[C@@]1(C)CO1
**62**	6.09 ± 0.06	0.82	6.67 ± 0.04	0.22	0.26	CC(C)C[C@H](NC(=O)[C@H](CC(C)C)NC(=O)[C@H](CC12CC3CC(CC(C3)C1)C2)N=[N+]=[N-])C(=O)N[C@@H](CC(C)C)C(=O)[C@@]1(C)CO1
**63**	6.08 ± 0.06	0.83	5.51 ± 0.00	3.12	3.72	CC(C)C[C@H](NC(=O)[C@H](Cc1ccccc1)NC(=O)[C@H](CC1CCCC2CCCCC12)NC(=O)[C@@H](Cc1ccccc1)N=[N+]=[N-])C(=O)[C@@]1(C)CO1
**64**	6.07 ± 0.14	0.85	6.55 ± 0.11	0.28	0.33	OC(=O)C(F)(F)F.COc1ccc(C[C@H](NC(=O)[C@H](CC2CCCCC2)NC(=O)CN2CCOCC2)C(=O)N[C@@H](CC(C)C)C(=O)[C@@]2(C)CO2)cc1
**65**	6.06 ± 0.28	0.87	7.88 ± 0.18	0.01	0.02	O=C(N[C@@H](CC1=CC=CC=C1)C(N[C@@H](CCS(CCNC(OC(C)(C)C)=O)(=O)=O)C(N[C@@H](CC(C)C)C([C@]2(CO2)C)=O)=O)=O)CC3=CC=CC=C3
**66**	6.05 ± 0.05	0.90	5.62 ± 0.10	2.41	2.69	[H][C@]1(CO1)C(=O)[C@H](CC1=CC=CC=C1)NC(=O)[C@H](CC1=CC=C(OC)C=C1)NC(=O)[C@H](C)NC(=O)C1=C(C)C2=C(C1)C=CC=C2
**67**	6.04 ± 0.08	0.91	6.28 ± 0.05	0.52	0.58	CC(C)C[C@H](NC(=O)CCCCCNC(=O)CCCCCNC(=O)CCCCCNC(=O)CC12CC3C4CC5CC3C(C1)C(C5)C4C2)C(=O)N[C@@H](CC(C)C)C(=O)N[C@@H](CC(C)C)C(=O)[C@@]1(C)CO1
**68**	6.03 ± 0.28	0.92	6.62 ± 0.18	0.24	0.26	O=C(N[C@H](CC1=CNC2=C1C=CC=C2)C(N[C@@H](CCCC)C(N[C@@H](CC(C)C)C([C@]3(CO3)C)=O)=O)=O)CCCCC
**69**	5.97 ± 0.10	1.07	5.42 ± 0.03	3.78	3.55	OC(=O)C(F)(F)F.CC(C)C[C@H](NC(=O)[C@H](Cc1ccc(CN)cc1)NC(=O)[C@@H](Cc1ccccc1)N=[N+]=[N-])C(=O)N[C@@H](CC1CCC(C)CC1)C(=O)[C@@]1(C)CO1
**70**	5.96 ± 0.10	1.09	6.58 ± 0.23	0.26	0.24	OC(=O)C(F)(F)F.COc1ccc(C[C@H](NC(=O)[C@H](Cc2ccc(cc2)-c2ccccc2)NC(=O)CN2CCOCC2)C(=O)N[C@@H](CC(C)C)C(=O)[C@@]2(C)CO2)cc1
**71**	5.94 ± 0.39	1.14	6.72 ± 0.07	0.19	0.17	O=C(N[C@@H](CC1=CNC2=C1C=CC=C2)C(N[C@@H](CC3=CNC4=C3C=CC=C4)C(N[C@@H](CC(C)C)C([C@]5(CO5)C)=O)=O)=O)CCCCC

**72**	5.93 ± 0.15	1.19	6.49 ± 0.05	0.32	0.28	CC(C)C[C@H](NC(=O)[C@H](Cc1ccccc1)NC(=O)[C@H](Cc1ccccc1)NC(=O)[C@@H](Cc1ccccc1)N=[N+]=[N-])C(=O)[C@@]1(C)CO1
**73** (Delanzomib)	5.92 ± 0.14	1.19	6.89 ± 0.06	0.13	0.11	CC(C)C[C@H](NC(=O)[C@@H](NC(=O)c1cccc(n1)-c1ccccc1)[C@@H](C)O)B(O)O
**74**	5.90 ± 0.30	1.26	6.79 ± 0.66	0.16	0.13	O=C([C@]1(C)OC1)[C@@H](NC([C@@H](NC([C@@H](NC(CCCCC)=O)CC2=CNC3=C2C=CC=C3)=O)CC4=CNC5=C4C=CC=C5)=O)CC6=C(C=CC=C7)C7=CC=C6
**75**	5.87 ± 0.21	1.34	5.02 ± 0.02	9.51	7.08	O=C(N[C@H](C(N[C@@H](CC1=C(F)C=C(F)C=C1)C([C@@]2(C)CO2)=O)=O)CCCC)C[C@H](CC(OC(C)(C)C)=O)NC(CCCCC)=O
**76**	5.87 ± 0.07	1.35	5.55 ± 0.09	2.84	2.09	CC[C@H](C)[C@H](NC(=O)CN=[N+]=[N-])C(=O)N[C@@H]([C@@H](C)CC)C(=O)N[C@@H]([C@@H](C)O)C(=O)N[C@@H](Cc1ccccc1)C(=O)[C@@]1(C)CO1
**77**	5.86 ± 0.70	1.37	5.63 ± 0.08	2.33	1.70	O=C([C@]1(C)OC1)[C@@H](NC([C@@H](NC([C@@H](NC(CCCCC)=O)CCC(OC(C)(C)C)=O)=O)CC2=CNC3=C2C=CC=C3)=O)CC4=CC=CC=C4
**78**	5.84 ± 0.29	1.43	6.55 ± 0.44	0.28	0.19	O=C([C@H](CCCC)NC([C@@H](NC(CCCCC)=O)C(C)C)=O)N[C@@H](CC1=CC=CC=C1)C=O
**79**	5.84 ± 0.24	1.46	6.61 ± 0.15	0.24	0.17	CC(C)C[C@H](NC(=O)[C@H](Cc1ccccc1)NC(=O)[C@H](Cc1ccc(C)cc1)NC(=O)[C@@H](Cc1ccccc1)N=[N+]=[N-])C(=O)[C@@]1(C)CO1
**80**	5.82 ± 0.16	1.51	5.24 ± 0.04	5.71	3.80	CCCCCC(=O)N[C@H](CC1=CN=CC=C1)C(=O)N[C@@H](CCCC)C(=O)N[C@@H](CC1=CC=CC=C1)C(=O)[C@@]1(C)CO1
**81**	5.81 ± 0.01	1.56	5.62 ± 0.01	2.38	1.55	CCC(C)[C@H](NC(=O)[C@H]([C@@H](C)CC)N(C)C(C)=O)C(=O)N[C@@H]([C@@H](C)O)C(=O)NC(CC(C)C)B1OC2[C@@H]3C[C@H](C[C@]2(C)O1)C3(C)C
**82**	5.80 ± 0.02	1.60	5.50 ± 0.02	3.14	2.00	CC(C)C[C@H](NC(=O)C(CC(C)C)NC(=O)[C@H](CC(C)C)NC(=O)OCc1ccccc1)\C=C\S(C)(=O)=O
**83**	5.78 ± 0.07	1.66	5.41 ± 0.09	3.90	2.34	O=C(N[C@H](C(N[C@@H](CC1=CC=CC=C1)C([C@@]2(C)CO2)=O)=O)CCCC)C[C@H](CC(OC(C)(C)C)=O)NC(CN3CCOCC3)=O
**84**	5.78 ± 0.24	1.67	8.08 ± 0.66	0.01	0.01	O=C(N[C@@H](C(C)C)C(N[C@@H](CCS(CCNC(OC(C)(C)C)=O)(=O)=O)C(N[C@@H](CC1=CC=CC=C1)C([C@]2(CO2)C)=O)=O)=O)CCCCC
**85**	5.78 ± 0.49	1.67	8.57 ± 0.33	0.003	<0.01	O=C(N[C@@H](CC(C)C)C([C@@]1(C)CO1)=O)[C@H](CCS(C2=CC=C(NC(OC(C)(C)C)=O)C=C2)(=O)=O)NC([C@H](C(C)C)NC(CCCCC)=O)=O
**86**	5.77 ± 0.23	1.71	5.72 ± 0.15	1.91	1.12	CCCCCC(N[C@H](C(N[C@H](C(N[C@@H](CC1=CC=CC=C1)B2OC3(C)C(CC4CC3C4(C)C)O2)=O)CC5=CNC6=C5C=CC=C6)=O)CC7=CNC8=C7C=CC=C8)=O
**87**	5.75 ± 0.04	1.76	5.25 ± 0.06	5.63	3.16	OC(=O)C(F)(F)F.CC(C)C[C@H](NC(=O)[C@H](Cc1ccc(CN)cc1)NC(=O)[C@@H](Cc1ccccc1)N=[N+]=[N-])C(=O)N[C@@H](CCC1CCCCC1)C(=O)[C@@]1(C)CO1

**88**	5.75 ± 0.09	1.79	5.26 ± 0.04	5.44	3.09	COc1ccc(C[C@H](NC(=O)[C@@H](C)NC(=O)C2=C(C)c3ccccc3C2)C(=O)N[C@@H](Cc2ccccc2)C(=O)[C@@]2(C)CO2)cc1
**89**	5.75 ± 0.15	1.77	5.52 ± 0.19	3.03	1.70	O=C(N[C@H](C(N[C@@H](CC1=C(F)C=C(F)C=C1)C([C@@]2(C)CO2)=O)=O)CCCC)C[C@H](CC(OC(C)(C)C)=O)NC(C3=COC=N3)=O
**90**	5.75 ± 0.14	1.77	5.76 ± 0.09	1.74	0.98	O=C([C@@]1(OC1)C)[C@H](CC2=CC=C(F)C=C2F)NC([C@H](CC3=CC=C(F)C=C3)NC([C@@H](CC(N4CCCC4)=O)NC([C@@H](N5CCCC5=O)CC6=CC=C(OC)C=C6)=O)=O)=O
**91**	5.71 ± 0.20	1.93	6.22 ± 0.15	0.61	0.31	CCCCCC(N[C@@H](CC1=CNC2=C1C=CC=C2)C(N[C@H](C(N[C@H](C([C@]3(OC3)C)=O)CC4=CC=CC=C4)=O)CC5=CNC6=C5C=CC=C6)=O)=O
**92**	5.71 ± 0.29	1.96	6.53 ± 0.30	0.30	0.15	CCC(C)[C@H](NC(C)=O)C(=O)N[C@@H](C(C)CC)C(=O)N[C@@H](COCC=C)C(=O)N[C@@H](CC(C)C)C(=O)[C@@]1(C)CO1
**93**	5.71 ± 0.51	1.96	7.70 ± 0.02	0.02	0.01	O=C([C@]1(C)OC1)[C@@H](NC([C@@H](NC([C@@H](NC(CC2CCCCC2)=O)CC3=CNC4=C3C=CC=C4)=O)CC5=CNC6=C5C=CC=C6)=O)CC7=CC=CC=C7
**94**	5.68 ± 0.03	2.07	5.18 ± 0.08	6.57	3.16	O=C(N[C@H](C(N[C@@H](CC1=CC=CC=C1)C([C@@]2(C)CO2)=O)=O)CCCC)C[C@@H](CCC(OC(C)(C)C)=O)NC(CCCCC)=O
**95**	5.62 ± 0.24	2.41	6.23 ± 0.05	0.59	0.25	O=C(N[C@@H](CC1=CC=C(NC(OC(C)(C)C)=O)C=C1)C(N[C@@H](CCCC)C(N[C@@H](CC(C)C)C([C@]2(CO2)C)=O)=O)=O)CCCCC
**96**	5.61 ± 0.11	2.44	5.15 ± 0.10	7.01	2.88	COc1ccc(C[C@H](NC(=O)[C@@H](C)NC(=O)CN2CCOCC2)C(=O)N[C@@H](Cc2ccc(cc2)-c2ccccc2)C(=O)[C@@]2(C)CO2)cc1
**97**	5.59 ± 0.33	2.58	6.95 ± 0.57	0.11	0.04	CCCCCC(=O)N[C@H](CCC(=O)OC(C)(C)C)C(=O)N[C@@H](CCCC)C(=O)N[C@H](CC1=CC=CC=C1)C(=O)[C@@]1(C)CO1
**98**	5.57 ± 0.12	2.72	5.15 ± 0.11	7.08	2.63	CC(C)C[C@H](NC(=O)OCc1ccccc1)C(=O)N[C@@H](CC(C)C)C(=O)N[C@@H](CC(C)C)C(=O)C(C)=O
**99**	5.56 ± 0.21	2.79	5.78 ± 0.06	1.67	0.60	CC(C)C[C@H](NC(=O)[C@H](CC(C)C)NC(=O)[C@H](CC(C)C)NC(=O)OCc1ccccc1)C=O
**100**	5.55 ± 0.17	2.81	5.99 ± 0.10	1.02	0.36	CCCCCC(=O)N[C@@H](CC1=CN=CC=C1)C(=O)N[C@@H](CCCC)C(=O)N[C@@H](CC1=CC=CC=C1)C(=O)[C@@]1(C)CO1
**101**	5.55 ± 0.07	2.85	6.44 ± 0.06	0.36	0.13	CCC(C)[C@H](NC(C)=O)C(=O)N[C@@H](C(C)CC)C(=O)N[C@@H](CC=C)C(=O)N[C@@H](CC(C)C)C(=O)[C@@]1(C)CO1
**102**	5.53 ± 0.14	2.92	6.53 ± 0.05	0.30	0.10	CC(C)C[C@H](NC(=O)[C@H](CC(C)C)NC(=O)[C@H](Cc1ccccc1)N=[N+]=[N-])C(=O)N[C@@H](CC(C)C)C(=O)[C@@]1(C)CO1
**103**	5.53 ± 0.31	2.96	7.37 ± 0.85	0.04	0.01	O=C(N[C@@H](CC1=CC=C(NC(OC(C)(C)C)=O)C=C1)C(N[C@@H](CC2=CC=C(NC(OC(C)(C)C)=O)C=C2)C(N[C@@H](CC3=CC=CC=C3)C([C@]4(CO4)C)=O)=O)=O)CCCCC
**104**	5.52 ± 0.12	2.99	5.50 ± 0.16	3.13	1.05	COC1=CC=C(C[C@H](NC(=O)[C@H](C)NC(=O)C2=C(C)C3=C(C2)C=CC=C3)C(=O)N[C@@H](CC2=CC=CC=C2)C(=O)C(C)=C)C=C1

**105**	5.52 ± 0.18	3.04	8.14 ± 1.02	0.01	<0.01	CCCCCC(N[C@H](C(N[C@H](C(N[C@@H](CC(C)C)B1OC2(C)C(CC3CC2C3(C)C)O1)=O)CC4=CNC5=C4C=CC=C5)=O)CC6=CNC7=C6C=CC=C7)=O
**106**	5.51 ± 0.00	3.11	6.58 ± 0.09	0.26	0.09	COc1ccc(C[C@H](NC(=O)[C@H](c2ccc3ccccc3c2)n2cc(CNC(=O)C3CC4CC3C=C4)nn2)C(=O)N[C@@H](Cc2ccccc2)C(=O)N[C@@H](CC(C)C)\C=C\S(C)(=O)=O)cc1
**107**	5.47 ± 0.36	3.42	6.61 ± 0.12	0.25	0.07	O=C(N[C@@H](CC1=CC=C(N)C=C1)C(N[C@@H](CCCC)C(N[C@@H](CC(C)C)C([C@]2(CO2)C)=O)=O)=O)CCCCC
**108**	5.46 ± 0.19	3.49	6.56 ± 0.04	0.27	0.08	CC(C)C[C@H](NC(=O)CC12CC3CC(CC(C3)C1)C2)C(=O)N[C@@H](CC(C)C)C(=O)N[C@@H](CC(C)C)C(=O)[C@@]1(C)CO1
**109** (Carfilzomib)	5.44 ± 0.11	3.66	5.92 ± 0.30	1.19	0.33	CC(C)C[C@@H](C(=O)[C@]1(CO1)C)NC(=O)[C@H](CC2=CC=CC=C2)NC(=O)[C@H](CC(C)C)NC(=O)[C@H](CCC3=CC=CC=C3)NC(=O)CN4CCOCC4
**110**	5.41 ± 0.00	3.87	<5.00	>10	>2.57	[H][C@]12CS[C@@H](CCCCC(=O)N[C@@H]([C@@H](C)CC)C(=O)N[C@@H]([C@@H](C)CC)C(=O)N[C@@H]([C@@H](C)O)C(=O)N[C@@H](CC(C)C)C(=O)[C@@]3(C)CO3)[C@@]1([H])NC(=O)N2
**111**	5.39 ± 0.15	4.05	5.67 ± 0.06	2.12	0.52	O=C([C@H](CCCC)NC([C@H](NC(CCCCC)=O)C(C)C)=O)N[C@@H](CC1=CC=CC=C1)/C=C/S(=O)(C)=O
**112**	5.39 ± 0.20	4.05	8.36 ± 0.87	0.004	<0.01	O=C(N[C@@H](C(C)C)C(N[C@@H](CCC(N[C@H](C([C@@]1(C)CO1)=O)CC(C)C)=O)C(N[C@@H](CC(C)C)C([C@]2(CO2)C)=O)=O)=O)CC3=CC=C(NC(OC(C)(C)C)=O)C=C3
**113**	5.35 ± 0.08	4.44	5.01 ± 0.01	9.72	2.19	OC(=O)C(F)(F)F.COC1CCC(C[C@H](NC(=O)[C@H](CC(C)C)NC(=O)[C@H](Cc2ccc(CN)cc2)NC(=O)[C@@H](Cc2ccccc2)N=[N+]=[N-])C(=O)[C@@]2(C)CO2)CC1
**114**	5.35 ± 0.08	4.50	5.27 ± 0.07	5.40	1.20	CCCCCC(=O)N[C@H](CC1=CC=NC=C1)C(=O)N[C@@H](CCCC)C(=O)N[C@@H](CC1=CC=CC=C1)C(=O)[C@@]1(C)CO1
**115**	5.35 ± 0.28	4.48	7.37 ± 0.10	0.04	0.01	O=C([C@H](CCCC)NC([C@@H](NC(CCCCC)=O)C(C)C)=O)N[C@@H](CC(C)C)B1OC2(C)C(CC3CC2C3(C)C)O1
**116**	5.34 ± 0.11	4.53	5.49 ± 0.09	3.26	0.71	C[C@@]1(C([C@@H](NC([C@H](CC2=CC=C(F)C=C2)NC([C@H](NC(CCCCC)=O)CC(N(CC)CC)=O)=O)=O)CC3=C(F)C=C(F)C=C3)=O)OC1
**117**	5.34 ± 0.12	4.55	6.32 ± 0.53	0.48	0.10	O=C([C@H](NC([C@@H](NC(CCC1=CC=CC=C1)=O)C(C)C)=O)CSC2=C(C(F)=C(C(F)=C2F)F)F)N[C@H](C([C@@]3(OC3)C)=O)CC(C)CO=C([C@H](NC([C@@H](NC(CCC1=CC=CC=C1)=O)C(C)C)=O)CSC2=C(C(F)=C(C(F)=C2F)F)F)N[C@H](C([C@@]3(OC3)C)=O)CC(C)C
**118**	5.33 ± 0.07	4.64	5.38 ± 0.09	4.20	0.89	CCCCCC(=O)N[C@H](C(C)C)C(=O)N[C@@H](CCCC)C(=O)N[C@@H](CC1=CC=CC=C1)C(=O)[C@@]1(C)CO1
**119**	5.28 ± 0.14	5.24	6.61 ± 0.10	0.24	0.05	O=C(N[C@@H](CC1=CC=NC=C1)C(N[C@@H](CCCC)C(N[C@@H](CC(C)C)C([C@]2(CO2)C)=O)=O)=O)CCCCC

**120**	5.27 ± 0.19	5.39	5.00 ± 0.00	> 10	1.86	COc1ccc(C[C@H](NC(=O)Cc2ccc3ccccc3c2)C(=O)N[C@@H](Cc2ccccc2)C(=O)N[C@@H](CC(C)C)\C=C\S(C)(=O)=O)cc1
**121**	5.25 ± 0.00	5.68	5.17 ± 0.07	6.73	1.20	COc1ccc(C[C@H](NC(=O)[C@@H](C)NC(=O)C2=C(C)c3ccccc3C2)C(=O)N[C@@H](Cc2ccc(cc2)-c2ccccc2)C(=O)[C@@]2(C)CO2)cc1
**122**	5.21 ± 0.04	6.11	5.00 ± 0.00	> 10	1.62	CCCC[C@H](NC(=O)C1CCCN1C(=O)[C@H](C)NC(=O)CCc1c(C)c2C=C3C=CC(c4ccc(OCCCN=[N+]=[N-])cc4)=[N]3[B-](F)(F)n2c1C)C(=O)N[C@@H](CC(C)C)C(=O)[C@@]1(C)CO1
**123**	5.20 ± 0.09	6.30	5.06 ± 0.05	8.70	1.38	COC1=CC=C(C[C@H](NC(=O)[C@H](C)NC(=O)C2=C(C)C3=C(C2)C=CC=C3)C(=O)N[C@@H](CC2=CC=CC=C2)C(=O)C=C)C=C1
**124**	5.20 ± 0.11	6.30	5.90 ± 0.00	1.26	0.20	CC(C)C[C@H](NC(=O)[C@H](CC1CCCCC1)NC(=O)[C@H](CC1CCCCC1)NC(=O)c1cnc(C)s1)C(=O)[C@@]1(C)CO1
**125**	5.20 ± 0.08	6.38	5.93 ± 0.06	1.17	0.19	CC[C@H](C)[C@H](NC(=O)CCC1=C(C)C2=Cc3ccc(-c4ccc(OCCCN=[N+]=[N-])cc4)n3[B](F)(F)[N+]2=C1C)C(=O)N[C@@H]([C@@H](C)CC)C(=O)N[C@@H]([C@@H](C)O)C(=O)N[C@@H](CC(C)C)C(=O)[C@@]1(C)CO1
**126**	5.20 ± 0.07	6.36	6.75 ± 0.08	0.18	0.03	O=C(N[C@@H](CC1=CC=C(N)C=C1)C(N[C@@H](CC2=CC=C(N)C=C2)C(N[C@@H](CC(C)C)C([C@]3(CO3)C)=O)=O)=O)CCCCC
**127**	5.19 ± 0.08	6.53	5.00 ± 0.00	9.90	1.55	CC(C)C[C@H](NC(=O)[C@H](Cc1ccccc1)NC(=O)[C@H](CC1CCC(CC1)C1CCCCC1)NC(=O)[C@@H](Cc1ccccc1)N=[N+]=[N-])C(=O)[C@@]1(C)CO1
**128**	5.19 ± 0.06	6.40	5.39 ± 0.02	4.07	0.63	O=C([C@H](CCCC)NC([C@H](NC(CCCCC)=O)C(C)C)=O)N[C@@H](CC(C)C)C=O
**129**	5.19 ± 0.08	6.44	6.07 ± 0.07	0.85	0.13	C[C@@]1(C([C@@H](NC([C@H](CCCC)NC([C@@H](NC(CCCCC)=O)CC2=CC=CN=C2)=O)=O)CC(C)C)=O)OC1
**130**	5.18 ± 0.03	6.66	5.14 ± 0.02	7.23	1.10	O=C(N[C@H](C(N[C@@H](CC1=CC=CC=C1)C([C@@]2(C)CO2)=O)=O)CCCC)C[C@H](CC(OC(C)(C)C)=O)NC(C3=COC=N3)=O
**131**	5.17 ± 0.03	6.71	<5.00	>10	>1.48	CC(C)C[C@H](NC(=O)[C@H](CC(C)C)N(C)C(=O)[C@H](CC(C)C)NC(=O)[C@H](Cc1ccccc1)N=[N+]=[N-])\C=C\S(C)(=O)=O
**132**	5.15 ± 0.12	7.02	<5.00	>10	>1.41	O=C(N[C@@H](C(C)C)C(N[C@@H](CCC(N[C@H](C([C@@]1(C)CO1)=O)CC(C)C)=O)C(O)=O)=O)CCCCC
**133**	5.13 ± 0.06	7.39	5.22 ± 0.05	6.06	0.81	CC[C@H](C)[C@H](NC(=O)CN=[N+]=[N-])C(=O)N[C@@H]([C@@H](C)CC)C(=O)N[C@@H]([C@@H](C)O)C(=O)N[C@@H](CC1CCCCC1)C(=O)[C@@]1(C)CO1
**134**	5.13 ± 0.08	7.48	8.86 ± 0.56	0.001	<0.01	O=C(N[C@@H](CC(C)C)C([C@@]1(C)CO1)=O)[C@H](CCS(C2=CC=C(N)C=C2)(=O)=O)NC([C@H](C(C)C)NC(CCCCC)=O)=O
**135**	5.12 ± 0.03	7.58	<5.00	>10	>1.32	O=C(N[C@H](C(N[C@@H](CC1=C(F)C=C(F)C=C1)C([C@@]2(C)CO2)=O)=O)CCCC)C[C@H](CC(OC(C)(C)C)=O)NC(CN3CCOCC3)=O

**136**	5.12 ± 0.08	7.61	<5.00	>10	>1.32	OC(=O)C(F)(F)F.CC(C)C[C@H](NC(=O)[C@H](Cc1ccc(CN)cc1)NC(=O)[C@@H](Cc1ccccc1)N=[N+]=[N-])C(=O)N[C@@H](CC1CC2CCC1C2)C(=O)[C@@]1(C)CO1
**137**	5.12 ± 0.05	7.66	6.07 ± 0.07	0.85	0.11	C[C@@]1(C([C@@H](NC([C@H](CC2=CC=C(F)C=C2)NC([C@@H](NC(CCC3=CC=CC=C3)=O)CC(N(CC)CC)=O)=O)=O)CC4=C(F)C=C(F)C=C4)=O)OC1
**138**	5.12 ± 0.06	7.65	6.76 ± 0.37	0.17	0.02	C[C@@]1(C([C@@H](NC([C@H](CCCC)NC([C@H](NC(COCOC)=O)C(C)C)=O)=O)CC2=CC=CC=C2)=O)OC1
**139**	5.11 ± 0.04	7.79	5.26 ± 0.04	5.47	0.71	C[C@@]1(C([C@@H](NC([C@H](CCCC)NC([C@H](NC(CCCCC)=O)CC2=CC=CC3=C2C=CC=C3)=O)=O)CC4=CC=CC=C4)=O)OC1
**140**	5.11 ± 0.05	7.83	5.77 ± 0.00	1.71	0.22	C[C@@]1(C([C@@H](NC([C@H](CCCC)NC([C@H](NC(CCCCC)=O)CC2=CNC3=C2C=CC=C3)=O)=O)CC4=CC=CC=C4)=O)OC1
**141**	5.10 ± 0.04	7.99	5.20 ± 0.05	6.24	0.79	C[C@@]1(C([C@@H](NC([C@H](CCCC)NC([C@H](NC(CCCCC)=O)CCC(N)=O)=O)=O)CC2=CC=CC=C2)=O)OC1
**142**	5.10 ± 0.04	7.96	5.27 ± 0.08	5.33	0.68	C[C@@]1(C([C@@H](NC([C@H](CCCC)NC([C@@H](NC(CCCCCCC)=O)C(C)C)=O)=O)CC2=CC=CC=C2)=O)OC1
**143**	5.09 ± 0.03	8.22	5.10 ± 0.09	7.93	0.98	C[C@@]1(C([C@@H](NC([C@H](CCCC)NC([C@@H](NC(CC2CCCC2)=O)C(C)C)=O)=O)CC3=CC=CC=C3)=O)OC1
**144**	5.09 ± 0.05	8.19	6.48 ± 0.12	0.33	0.04	C[C@@]1(C([C@@H](NC([C@H](CCCC)NC([C@@H](NC(CCCCC)=O)CC2=CC=CC=N2)=O)=O)CC3=CC=CC=C3)=O)OC1
**145**	5.09 ± 0.04	8.10	6.52 ± 0.28	0.30	0.04	O=C(N[C@@H](CC1=CC=CC=C1)C(N[C@@H](CCCC)C(N[C@@H](CC2=CC=CC=C2)C([C@]3(CO3)C)=O)=O)=O)CCCCC
**146**	5.08 ± 0.04	8.29	5.30 ± 0.02	5.00	0.60	C[C@@]1(C([C@@H](NC([C@H](CCCC)NC([C@@H](NC(CC2=CC=CC=C2)=O)C(C)C)=O)=O)CC3=CC=CC=C3)=O)OC1
**147**	5.08 ± 0.05	8.36	5.66 ± 0.08	2.17	0.26	O=C(N[C@@H](CC1=CC=CC=C1)C(N[C@@H](CCCC)C(N[C@@H](CC(C)C)C([C@]2(CO2)C)=O)=O)=O)CCCCC
**148**	5.07 ± 0.03	8.57	5.01 ± 0.01	9.66	1.15	O=C([C@H](CCCC)NC([C@H](NC(CCCCC)=O)C(C)C)=O)N[C@@H](CC(C)C)/C=C/C(C)=O
**149**	5.07 ± 0.03	8.47	5.30 ± 0.00	5.06	0.59	CC[C@H](C)[C@H](NC(=O)Cn1cc(CNC(=O)CCCCC[N+]2([Cl-])=C(\C=C\C=C\C=C3\N(C)c4ccccc4C3(C)C)C(C)(C)c3ccccc23)nn1)C(=O)N[C@@H]([C@@H](C)CC)C(=O)N[C@@H]([C@@H](C)O)C(=O)N[C@@H](CC(C)C)C(=O)[C@@]1(C)CO1
**150**	5.07 ± 0.05	8.42	5.61 ± 0.04	2.43	0.29	CC[C@H](C)[C@H](NC(=O)CN=[N+]=[N-])C(=O)N[C@@H]([C@@H](C)CC)C(=O)N[C@@H]([C@@H](C)O)C(=O)N[C@@H](CCC1CCCCC1)C(=O)[C@@]1(C)CO1
**151**	5.07 ± 0.03	8.45	6.52 ± 0.33	0.30	0.04	O=C(N[C@@H](CC1=CC=C(NC(OC(C)(C)C)=O)C=C1)C(N[C@@H](CCC(N[C@H](C([C@@]2(C)CO2)=O)CC(C)C)=O)C(N[C@@H](CC(C)C)C([C@]3(CO3)C)=O)=O)=O)CCCCC
**152**	5.07 ± 0.07	8.50	7.32 ± 0.12	0.05	0.01	O=C(N[C@@H](CC1=CC=C(NC(OC(C)(C)C)=O)C=C1)C(N[C@@H](CC2=CC=C(NC(OC(C)(C)C)=O)C=C2)C(N[C@@H](CC(C)C)C([C@]3(CO3)C)=O)=O)=O)CCCCC

**153**	5.07 ± 0.04	8.52	7.38 ± 0.26	0.04	<0.01	O=C(N[C@@H](CC1=CNC2=C1C=CC=C2)C(N[C@@H](CCCC)C(N[C@@H](CC(C)C)C([C@]3(CO3)C)=O)=O)=O)CCCCC
**154**	5.06 ± 0.02	8.62	5.01 ± 0.01	9.73	1.12	C[C@@]1(C([C@@H](NC([C@H](CC2=CC=C(F)C=C2)NC([C@H](NC(CCC3=CC=CC=C3)=O)CC(N(CC)CC)=O)=O)=O)CC4=C(F)C=C(F)C=C4)=O)OC1
**155**	5.06 ± 0.03	8.76	5.11 ± 0.05	7.84	0.89	O=C([C@H](CCCC)NC([C@H](NC(CCCCC)=O)C(C)C)=O)N[C@@H](CC1=CC=CC=C1)/C=C/C(C)=O
**156**	5.06 ± 0.03	8.71	5.14 ± 0.06	7.31	0.83	COC1=CC=C(C[C@H](NC(=O)[C@H](C)NC(=O)CN2CCOCC2)C(=O)N[C@@H](CC2CCCCC2)C(=O)[C@@]2(C)CO2)C=C1
**157**	5.06 ± 0.05	8.66	5.58 ± 0.41	2.64	0.30	O=C(N[C@@H](CC1=CC=C(NC(OC(C)(C)C)=O)C=C1)C(N[C@@H](CCCC)C(N[C@@H](CC2=CC=CC=C2)C([C@]3(CO3)C)=O)=O)=O)CCCCC
**158**	5.06 ± 0.05	8.78	6.18 ± 0.49	0.67	0.08	O=C([C@]1(C)OC1)[C@@H](NC([C@@H](NC([C@@H](NC(CCCCC)=O)CC2=CNC3=C2C=CC=C3)=O)CC4=CNC5=C4C=CC=C5)=O)CC6=C(F)C=C(F)C=C6
**159**	5.06 ± 0.03	8.68	7.71 ± 0.15	0.02	0.000	O=C(N[C@@H](CC1=CNC2=C1C=CC=C2)C(N[C@@H](CCCC)C(N[C@@H](CC3=CC=CC=C3)C([C@]4(CO4)C)=O)=O)=O)CCCCC
**160**	5.05 ± 0.04	8.93	<5.00	>10	>1.12	C[C@@H](NC(=O)C1=C(C)c2ccccc2C1)C(=O)N[C@@H](Cc1ccc(OCCN=[N+]=[N-])cc1)C(=O)N[C@@H](CC1CCCCC1)C(=O)[C@@]1(C)CO1
**161**	5.05 ± 0.03	8.83	5.22 ± 0.05	5.96	0.68	O=C([C@H](CCCC)NC([C@@H](NC(CCCCC)=O)C(C)C)=O)N[C@@H](CC1=CC=CC=C1)/C=C\S(C)(=O)=O
**162**	5.05 ± 0.04	8.96	5.36 ± 0.06	4.32	0.49	OC(=O)C(F)(F)F.CC(C)C[C@H](NC(=O)[C@H](Cc1ccc(CN)cc1)NC(=O)[C@@H](Cc1ccccc1)N=[N+]=[N-])C(=O)N[C@@H](CC1CCC(CC1)C1CCCCC1)C(=O)[C@@]1(C)CO1
**163**	5.05 ± 0.03	9.01	6.21 ± 0.04	0.62	0.07	O=C(N[C@@H](C(C)C)C(N[C@@H](CCCC)C(N[C@@H](CC(C)C)C([C@]1(CO1)C)=O)=O)=O)CCCCC
**164**	5.04 ± 0.03	9.13	<5.00	>10	>1.1	CCCC[C@H](NC(=O)[C@@H]1CCN1C(=O)[C@H](C)NC(=O)CN=[N+]=[N-])C(=O)N[C@@H](CC(C)C)C(=O)[C@@]1(C)CO1
**165**	5.04 ± 0.03	9.23	<5.00	>10	>1.1	CC(C)C[C@H](NC(=O)[C@H](CC(C)C)n1cc(C)cc(NC(=O)c2cnccn2)c1=O)C(=O)[C@@]1(C)CO1
**166**	5.04 ± 0.04	9.02	5.13 ± 0.05	7.35	0.81	O=C([C@H](CCCC)NC([C@H](NC(CCCCC)=O)C(C)C)=O)N[C@@H](CC1=CC=CC=C1)/C=C\S(C)(=O)=O
**167**	5.04 ± 0.03	9.05	5.33 ± 0.03	4.69	0.51	CC(C)C[C@H](NC(=O)[C@H](CC(C)C)NC(=O)[C@H](CC(C)C)NC(=O)CCCCCNC(=O)CCCCCNC(=O)CCCCCNC(=O)CC12CC3C[C@@H](CC(C3)C1)C2)\C=C\S(C)(=O)=O
**168**	5.04 ± 0.03	9.08	5.37 ± 0.04	4.24	0.47	O=C([C@H](CCCC)NC([C@@H](NC(CCCCC)=O)C(C)C)=O)N[C@@H](CC(C)C)/C=C/S(=O)(C)=O

**169**	5.03 ± 0.02	9.32	<5.00	>10	>1.07	CC(C)CC(NC(=O)[C@H](CC(C)C)NC(=O)C(CC(C)C)NC(=O)CCCCCNC(=O)CCCCCNC(=O)CCCCCNC(=O)CC12CC3C[C@@H](CC(C3)C1)C2)B1OC2[C@@H]3C[C@H](C[C@]2(C)O1)C3(C)C
**170**	5.03 ± 0.02	9.37	<5.00	>10	>1.07	C[C@H](NC(=O)CN1CCOCC1)C(=O)N[C@@H](Cc1ccc(OCc2cn(CCCC3=C4C(C)=CC(C)=[N]4[B](F)(F)C4C(C)=CC(C)=C34)nn2)cc1)C(=O)N[C@@H](CC1CCCC2CCCCC12)C(=O)[C@@]1(C)CO1
**171**	5.03 ± 0.02	9.41	<5.00	>10	>1.07	CC(C)C[C@H](NC(=O)[C@H](Cc1ccc(CN)cc1)NC(=O)[C@H](Cc1ccccc1)N=[N+]=[N-])C(=O)N[C@@H](Cc1ccc(N)cc1)\C=C\S(C)(=O)=O
**172**	5.02 ± 0.02	9.45	<5.00	>10	>1.05	O=C(N[C@@H](C(C)C)C(N[C@@H](CCC(N[C@H](C([C@@]1(C)CO1)=O)CC(C)C)=O)C(NC(CCS(=O)(C2=CC=C(NC(OC(C)(C)C)=O)C=C2)=O)C(OC)=O)=O)=O)CCCCC
**173**	5.02 ± 0.01	9.62	<5.00	>10	>1.05	COc1ccc(C[C@H](NC(=O)[C@H](C)NC(=O)CN2CCOCC2)C(=O)N[C@@H](CC2CC3CCC2C3)C(=O)[C@@]2(C)CO2)cc1
**174**	5.02 ± 0.01	9.64	<5.00	>10	>1.05	FC(F)(F)C=O.CC(C)C[C@H](NC(=O)[C@H](Cc1ccccc1)N=[N+]=[N-])C(=O)N[C@@H](CC(C)(C)F)C(=O)N[C@@H](Cc1ccc(CN)cc1)\C
**175**	5.02 ± 0.02	9.44	5.06 ± 0.04	8.61	0.91	O=C(CCCCC)N[C@@H](C(C)C)C(N[C@@H](CCS(=O)(C1=CC=C(NC(NCC#C)=O)C=C1)=O)C(N[C@@H](CC(C)C)C([C@]2(C)OC2)=O)=O)=O
**176**	5.02 ± 0.02	9.44	5.70 ± 0.14	1.98	0.21	O=C(N[C@@H](CC1=CC=C(N)C=C1)C(N[C@@H](CCCC)C(N[C@@H](CC2=CC=CC=C2)C([C@]3(CO3)C)=O)=O)=O)CCCCC
**177**	5.01 ± 0.01	9.75	<5.00	>10	>1.02	FC(F)(F)C=O.CC(C)C[C@H](NC(=O)[C@H](Cc1ccccc1)N=[N+]=[N-])C(=O)N[C@@H](CO)C(=O)N[C@@H](Cc1ccc(CN)cc1)\C=C\S(C
**178**	5.01 ± 0.01	9.74	5.01 ± 0.01	9.74	1.00	OC(=O)C(F)(F)F.CC(C)C[C@H](NC(=O)[C@H](Cc1ccc(CN)cc1)NC(=O)[C@@H](Cc1ccccc1)N=[N+]=[N-])C(=O)N[C@@H](C1CCCCC1)C(=O)[C@@]1(C)CO1
**179**	5.01 ± 0.01	9.82	<5.00	>10	>1.02	C[C@@H](NC(=O)C1=C(C)c2ccccc2C1)C(=O)N[C@@H](Cc1c[nH]c2ccccc12)C(=O)N[C@@H](CC1CCCCC1)C(=O)[C@@]1(C)CO1
**180**	5.01 ± 0.01	9.86	<5.00	>10	>1.02	O=C([C@H](CCCC)NC([C@@H](NC(CCCCC)=O)C(C)C)=O)N[C@@H](CC(C)C)C(C(C)=C)=O
**181**	5.01 ± 0.00	9.85	<5.00	>10	>1.02	FC(F)(F)C=O.CC(C)C[C@H](NC(=O)[C@H](CC(C)C)NC(=O)[C@H](CCN)NC(=O)[C@H](Cc1ccccc1)N=[N+]=[N-])\C=C\S(C)(=O)=O
**182**	5.01 ± 0.00	9.85	5.26 ± 0.10	5.54	0.56	OC(=O)C(F)(F)F.CC(C)C[C@H](NC(=O)[C@H](Cc1ccc(CN)cc1)NC(=O)[C@@H](Cc1ccccc1)N=[N+]=[N-])C(=O)N[C@@H](CC1CCC2CCCCC2C1)C(=O)[C@@]1(C)CO1

II. Inactive against *T. vaginalis* , active against HeLa

**183**	<5.00	>10	6.39 ± 0.20	0.41	<0.04	CC(C)C[C@H](NC(=O)[C@H](Cc1ccccc1)NC(=O)[C@H](Cc1ccc(cc1)-c1ccccc1)NC(=O)[C@H](Cc1ccccc1)N=[N+]=[N-])C(=O)[C@@]1(C)CO1
**184**	<5.00	>10	5.94 ± 0.00	1.16	<0.11	CCCCCCCCCCNC(=O)N[C@@H](C(C)C)C(=O)N[C@@H](CC(C)C)C(=O)N[C@@H](CC(C)C)C(=O)[C@@]1(C)CO1
**185**	<5.00	>10	5.84 ± 0.10	1.46	<0.14	CC(C)C[C@H](NC(=O)OCc1ccccc1)C(=O)N[C@@H](CC(C)C)C(=O)N[C@@H](CC(C)C)C(=C)C(C)=O
**186**	<5.00	>10	5.52 ± 0.06	3.02	<0.30	OC(=O)C(F)(F)F.COc1ccc(C[C@H](NC(=O)[C@H](C)NC(=O)CN2CCOCC2)C(=O)N[C@@H](C[C@H]2CCCc3ccccc23)C(=O)[C@@]2(C)CO2)cc1
**187**	<5.00	>10	5.50 ± 0.00	3.13	<0.32	CC(C)C[C@H](NC(=O)OCc1ccccc1)C(=O)NC(CC(C)C)C(=O)N[C@@H](CC(C)C)C(=O)[C@@]1(C)CO1
**188**	<5.00	>10	5.37 ± 0.02	4.31	<0.43	COc1ccc(C[C@H](NC(=O)[C@@H](C)NC(=O)CN2CCOCC2)C(=O)N[C@@H](Cc2ccccc2)C(=O)[C@@]2(C)CO2)cc1
**189**	<5.00	>10	5.30 ± 0.03	5.04	<0.50	CCC(C)C(NC(=O)CN=[N+]=[N-])C(=O)NC(CO)C(=O)NC(C(C)O)C(=O)NC(CC(C)C)C(=O)C1(C)CO1
**190**	<5.00	>10	5.26 ± 0.04	5.52	<0.55	C[C@@H](NC(=O)CN1CCOCC1)C(=O)N[C@@H](Cc1c[nH]c2ccccc12)C(=O)N[C@@H](Cc1ccccc1)C(=O)[C@@]1(C)CO1
**191**	<5.00	>10	5.26 ± 0.08	5.54	<0.55	O=C(CNC(C1=C(C=CC(Cl)=C1)Cl)=O)N[C@H](C([C@@]2(OC2)C)=O)CC(C)C
**192**	<5.00	>10	5.26 ± 0.09	5.51	<0.55	OC(=O)C(F)(F)F.CC(C)C[C@H](NC(=O)CN1CCOCC1)C(=O)N[C@@H](C[C@H]1CC[C@@H](CC1)C1CCCCC1)C(=O)N[C@@H](Cc1ccccc1)C(=O)N[C@@H](C)C(=O)[C@@]1(C)CO1
**193**	<5.00	>10	5.24 ± 0.01	5.81	<0.58	C[C@H](NC(=O)[C@H](Cc1ccccc1)NC(=O)[C@H](C[C@H]1CC[C@@H](CC1)C1CCCCC1)NC(=O)[C@H](Cc1ccccc1)n1cc(CNC(=O)C2CC3CC2C=C3)nn1)C(=O)[C@@]1(C)CO1
**194**	<5.00	>10	5.24 ± 0.05	5.75	<0.58	COc1ccc(C[C@H](NC(=O)[C@H](C)NC(=O)CN2CCOCC2)C(=O)N[C@@H](CC2CCC(CC2)C2CCCCC2)C(=O)[C@]2(C)CO2)cc1
**195**	<5.00	>10	5.22 ± 0.09	5.99	<0.60	CC(C)C[C@H](NC(=O)[C@H](CC12CC3CC(CC(C3)C1)C2)NC(=O)[C@H](Cc1ccccc1)N=[N+]=[N-])C(=O)N[C@@H](CC(C)C)C(=O)[C@@]1(C)CO1
**196**	<5.00	>10	5.19 ± 0.11	6.52	<0.65	OC(=O)C(F)(F)F.CC(C)C[C@H](NC(=O)[C@H](Cc1ccc(CN)cc1)NC(=O)[C@@H](Cc1ccccc1)N=[N+]=[N-])C(=O)N[C@@H](CC1CCCC2CCCCC12)C(=O)[C@@]1(C)CO1
**197**	<5.00	>10	5.15 ± 0.07	7.05	<0.71	OC(=O)C(F)(F)F.COc1ccc(C[C@H](NC(=O)[C@H](C)NC(=O)CN2CCOCC2)C(=O)N[C@@H](CC2CC[C@@H](CC2)C2CCCCC2)C(=O)[C@@]2(C)CO2)cc1

**198**	<5.00	>10	5.14 ± 0.06	7.22	<0.72	CCC(C)[C@H](NC(=O)CCc1c(C)c2C=C3C=CC(c4ccc(OCCCn5cc(CNC(=O)CCCC[C@@H]6SCC7NC(=O)NC67)nn5)cc4)=[N]3[B](F)(F)n2c1C)C(=O)N[C@@H]([C@@H](C)CC)C(=O)N[C@@H]([C@H](C)O)C(=O)N[C@@H](CC(C)C)[C@]1(CO1)C(C)=O
**199**	<5.00	>10	5.14 ± 0.06	7.32	<0.72	OC(=O)C(F)(F)F.CC(C)C[C@H](NC(=O)[C@H](Cc1ccccc1)n1cc(CCCCC2=C3C(C)=CC(C)=[N]3[B](F)(F)n3c(C)cc(C)c23)nn1)C(=O)N[C@@H](C)C(=O)N[C@@H](Cc1ccc(CN)cc1)\C=C\S(C)(=O)=O
**200**	<5.00	>10	5.14 ± 0.00	7.29	<0.72	CC(C)C[C@H](NC(=O)[C@H](Cc1ccccc1)NC(=O)[C@H](CC(C)C)NC(=O)[C@H](CCc1ccccc1)NC(=O)CN1CCOCC1)\C=C\S(C)(=O)=O
**201**	<5.00	>10	5.14 ± 0.05	7.29	<0.72	OC(=O)C(F)(F)F.COc1ccc(C[C@H](NC(=O)[C@H](C)NC(=O)CN2CCOCC2)C(=O)N[C@@H](CC2CC[C@@H](CC2)c2ccccc2)C(=O)[C@@]2(C)CO2)cc1
**202**	<5.00	>10	5.14 ± 0.07	7.28	<0.72	C[C@@H](NC(=O)C1CC2CC1C=C2)C(=O)N[C@@H](Cc1c[nH]c2ccccc12)C(=O)N[C@@H](CC1CCCCC1)C(=O)C1(C)CO1
**203**	<5.00	>10	5.13 ± 0.09	7.40	<0.74	CC(C)C[C@H](NC(=O)[C@H](CC(C)C)NC(=O)[C@H](CC(C)C)NC(=O)CCCCCNC(=O)CCCCCNC(=O)CCCCCNS(=O)(=O)c1cccc2c(cccc12)N(C)C)\C=C\S(C)(=O)=O
**204**	<5.00	>10	5.10 ± 0.05	7.96	<0.79	OC(=O)C(F)(F)F.OC(=O)C(F)(F)F.CC(C)[C@H](NC(=O)OCc1ccccc1)C(=O)N[C@@H](CCCNC(N)=N)C(=O)N1CCC[C@H]1C(=O)N[C@@H](CCCNC(N)=N)C#C
**205**	<5.00	>10	5.09 ± 0.07	8.15	<0.81	C[C@@H](NC(=O)CN1CCOCC1)C(=O)N[C@@H](Cc1c[nH]c2ccccc12)C(=O)N[C@@H](CC1CCCCC1)C(=O)[C@@]1(C)CO1
**206**	<5.00	>10	5.08 ± 0.05	8.40	<0.83	OC(=O)C(F)(F)F.COC1CCC(C[C@H](NC(=O)c2cnc(C)s2)C(=O)N[C@@H](CC(C)C)C(=O)N[C@@H](Cc2ccc(CN)cc2)\C=C\S(C)(=O)=O)CC1
**207**	<5.00	>10	5.08 ± 0.03	8.36	<0.83	C[C@@H](NC(=O)C1=C(C)c2ccccc2C1)C(=O)N[C@@H](Cc1c[nH]c2ccccc12)C(=O)N[C@@H](Cc1ccc(cc1)-c1ccccc1)C(=O)[C@@]1(C)CO1
**208**	<5.00	>10	5.08 ± 0.06	8.34	<0.83	CCCC[C@H](NC(=O)[C@@H]1CC(F)(F)CN1C(=O)[C@H](C)NC(=O)CN=[N+]=[N-])C(=O)N[C@@H](CC1CCC(F)(F)CC1)C(=O)[C@@]1(C)CO1
**209**	<5.00	>10	5.08 ± 0.07	8.25	<0.83	CC[C@H](C)[C@H](NC(=O)CN=[N+]=[N-])C(=O)N[C@@H]([C@@H](C)CC)C(=O)N[C@@H]([C@@H](C)O)C(=O)N[C@@H](CCc1ccccc1)C(=O)[C@@]1(C)CO1
**210**	<5.00	>10	5.07 ± 0.03	8.60	<0.85	C[C@@]1(C([C@@H](NC([C@@H](CCCC)NC([C@H](NC(CCCCC)=O)CC2=CNC3=C2C=CC=C3)=O)=O)CC4=CC=CC=C4)=O)OC1
**211**	<5.00	>10	5.07 ± 0.06	8.50	<0.85	CC(C)C[C@H](NC(=O)[C@@H](NC(=O)N[C@H](C(C)C)C(=O)OC(C)(C)C)C(C)C)C(=O)[C@@]1(C)CO1

**212**	<5.00	>10	5.07 ± 0.06	8.48	<0.85	OC(=O)C(F)(F)F.CC(C)C[C@H](NC(=O)[C@H](CC1CCC2CCCCC2C1)NC(=O)c1cnc(C)s1)C(=O)N[C@@H](Cc1ccc(CN)cc1)\C=C\S(C)(=O)=O
**213**	<5.00	>10	5.07 ± 0.05	8.48	<0.85	OC(=O)C(F)(F)F.OC(=O)C(F)(F)F.CC(C)[C@H](NC(=O)OCc1ccccc1)C(=O)N[C@@H](CCCNC(N)=N)C(=O)N1CCC[C@H]1C(=O)N[C@@H](CCCNC(N)=N)C(=O)CF
**214**	<5.00	>10	5.07 ± 0.06	8.42	<0.85	C[C@@H](NC(=O)C1=C(C)c2ccccc2C1)C(=O)N[C@@H](Cc1ccc(OCCn2cc(CNC(=O)C3CC4CC3C=C4)nn2)cc1)C(=O)N[C@@H](CC1CCCCC1)C(=O)[C@@]1(C)CO1
**215**	<5.00	>10	5.06 ± 0.05	8.64	<0.87	OC(=O)C(F)(F)F.CC(C)C[C@H](NC(=O)c1cnc(C)s1)C(=O)N[C@@H](CC1CCC(CC1)C1CCCCC1)C(=O)N[C@@H](Cc1ccc(CN)cc1)\C=C\S(C)(=O)=O
**216**	<5.00	>10	5.05 ± 0.04	9.01	<0.89	[H][C@@]12CCCC[C@]1([H])[C@@H](C[C@H](NC(=O)[C@H](Cc1ccc(OC)cc1)NC(=O)[C@H](C)NC(=O)CN1CCOCC1)C(=O)[C@@]1(C)CO1)CCC2
**217**	<5.00	>10	5.05 ± 0.02	8.96	<0.89	CC(C)C[C@H](NN[C@@H](CC(C)C)C(=O)c1ccco1)C(=O)N[C@@H](CC(C)C)C(=O)N[C@H](Cc1ccccc1)C(=O)N=[N+]=[N-]
**218**	<5.00	>10	5.05 ± 0.04	8.94	<0.89	CC(C)C[C@H](NC(=O)[C@H](CC(C)C)n1cc(C)cc(NC(=O)c2cc(C)on2)c1=O)\C=C\S(C)(=O)=O
**219**	<5.00	>10	5.05 ± 0.04	8.91	<0.89	C[C@H](NC(=O)[C@H](Cc1ccccc1)NC(=O)[C@H](C[C@H]1CC[C@@H](CC1)C1CCCCC1)NC(=O)[C@@H](Cc1ccccc1)N=[N+]=[N-])C(=O)[C@@]1(C)CO1
**220**	<5.00	>10	5.05 ± 0.04	8.88	<0.89	FC(F)(F)C=O.CC(C)C[C@H](NC(=O)[C@H](Cc1ccccc1)N=[N+]=[N-
**221**	<5.00	>10	5.04 ± 0.03	9.22	<0.91	OC(=O)C(F)(F)F.C[C@@H](NC(=O)CN1CCOCC1)C(=O)N[C@@H](Cc1ccc(OCCn2cc(CNC(=O)C3CC4CC3C=C4)nn2)cc1)C(=O)N[C@@H](CC1CCCCC1)C(=O)[C@@]1(C)CO1
**222**	<5.00	>10	5.04 ± 0.03	9.20	<0.91	OC(=O)C(F)(F)F.[H][C@@]12CCCC[C@]1([H])[C@H](C[C@H](NC(=O)[C@H](Cc1ccc(OC)cc1)NC(=O)[C@H](C)NC(=O)CN1CCOCC1)C(=O)[C@@]1(C)CO1)CCC2
**223**	<5.00	>10	5.04 ± 0.03	9.18	<0.91	C[C@@]1(CO1)C(=O)[C@H](CCC1CCCCC1)NC(=O)[C@H](Cc1ccccc1)NC(=O)CNC(=O)CN1CCOCC1
**224**	<5.00	>10	5.04 ± 0.03	9.13	<0.91	OC(=O)C(F)(F)F.CC(C)C[C@H](NC(=O)c1cnc(C)s1)C(=O)N[C@@H](CC1CCC(C)CC1)C(=O)N[C@@H](Cc1ccc(CN)cc1)\C=C\S(C)(=O)=O
**225**	<5.00	>10	5.04 ± 0.02	9.08	<0.91	COc1ccc(cc1)C1=[N]2C(C(C)=C1)=C(CCCCc1cn(nn1)[C@@H](Cc1ccccc1)C(=O)N[C@H](C)C(=O)N[C@@H](Cc1c[nH]c3ccccc13)C(=O)N[C@@H](CC1CCCCC1)C(=O)[C@@]1(C)CO1)c1c(C)cc(-c3ccc(OC)cc3)n1[B-]2(F)F
**226**	<5.00	>10	5.04 ± 0.03	9.06	<0.91	OC(=O)C(F)(F)F.CC(C)C[C@H](NC(=O)c1cnc(C)s1)C(=O)N[C@@H](CC1CCC2CCCCC2C1)C(=O)N[C@@H](Cc1ccc(CN)cc1)\C=C\S(C)(=O)=O

**227**	<5.00	>10	5.04 ± 0.03	9.04	<0.91	COc1ccc(C[C@H](NC(=O)[C@@H](C)NC(=O)C2=C(C)c3ccccc3C2)C(=O)N[C@@H](CC2CCC(F)(F)CC2)C(=O)[C@@]2(C)CO2)cc1
**228**	<5.00	>10	5.04 ± 0.03	9.03	<0.91	CC(C)C[C@H](NC(=O)[C@H](CC(C)C)NC(=O)[C@H](CC(C)C)NC(=O)CCCCCNC(=O)CCCCCNC(=O)CCCCCNC(=O)CC12CC3C4CC5CC3C(C1)C(C5)C4C2)\C=C\S(C)(=O)=O
**229**	<5.00	>10	5.04 ± 0.02	9.03	<0.91	C[C@H](NC(=O)[C@H](Cc1ccccc1)NC(=O)[C@H](C[C@H]1CC[C@@H](CC1)c1ccccc1)NC(=O)[C@@H](Cc1ccccc1)N=[N+]=[N-])C(=O)[C@@]1(C)CO1
**230**	<5.00	>10	5.03 ± 0.02	9.42	<0.93	FC(F)(F)C=O.CC(C)C[C@H](NC(=O)[C@H](CC(C)C)NC(=O)c1cnc(C)s1)C(=O)N[C@@H](Cc1ccc(CN)cc1)\C=C\S(C)(=O)=O
**231**	<5.00	>10	5.03 ± 0.01	9.35	<0.93	CCCC[C@H](NC(=O)[C@@H]1CCCN1C(=O)[C@H](C)NC(=O)Cn1cc(CCCCC2=C3C(C)=CC(C)=[N]3[B-](F)(F)n3c(C)cc(C)c23)nn1)C(=O)N[C@@H](CC(C)C)C(=O)[C@@]1(C)CO1
**232**	<5.00	>10	5.03 ± 0.02	9.33	<0.93	CCCCCCCCCCNC(=O)N[C@@H](C(C)C)C(=O)N[C@@H](CC(C)C)C(=O)N[C@@H](CC(C)C)\C=C\S(C)(=O)=O
**233**	<5.00	>10	5.03 ± 0.03	9.27	<0.93	C[C@@]1(CO1)C(=O)[C@H](CC1CCCCC1)NC(=O)[C@H](Cc1ccccc1)NC(=O)CNC(=O)CN1CCOCC1
**234**	<5.00	>10	5.03 ± 0.03	9.26	<0.93	CC(C)C[C@H](NC(=O)[C@H](CC(C)C)NC(=O)[C@H](Cc1ccccc1)N=[N+]=[N-])C(=O)N[C@@H](Cc1cnc[nH]1)\C=C\S(C)(=O)=O
**235**	<5.00	>10	5.02 ± 0.02	9.57	<0.95	FC(F)(F)C=O.CC(C)C[C@H](NC(=O)[C@H](Cc1ccccc1)N=[N+]=[N-])C(=O)N[C@@H](C)C(=O)N[C@@H](CCCN)\C=C\S(C)(=O)=O
**236**	<5.00	>10	5.02 ± 0.02	9.56	<0.95	CC(C)C[C@H](NC(=O)OCc1ccccc1)C(=O)N[C@@H](CC(C)C)C(=O)NC(CC(C)C)P(=O)(Oc1ccccc1)Oc1ccccc1
**237**	<5.00	>10	5.02 ± 0.02	9.49	<0.95	COc1ccc(C[C@H](NC(=O)[C@H](C)NC(=O)CN2CCOCC2)C(=O)N[C@@H](CC2CCCCC2)\C=C\S(C)(=O)=O)cc1
**238**	<5.00	>10	5.02 ± 0.02	9.48	<0.95	CC(C)C[C@H](NC(=O)[C@H](CC(C)C)NC(=O)[C@H](CC(C)C)NC(=O)CN=[N+]=[N-])\C=C\S(C)(=O)=O
**239**	<5.00	>10	5.01 ± 0.01	9.79	<0.98	OC(=O)C(F)(F)F.CC(C)C[C@H](NC(=O)[C@H](CC(C)C)NC(=O)[C@@H](Cc1ccccc1)N=[N+]=[N-])C(=O)N[C@@H](Cc1ccc(CN)cc1)\C=C\S(C)(=O)=O
**240**	<5.00	>10	5.01 ± 0.01	9.70	<0.98	OC(=O)C(F)(F)F.CC(C)CC(C(=O)N[C@@H](Cc1ccc(CN)cc1)\C=C\S(C)(=O)=O)n1cc(C)cc(NC(=O)OCc2ccccc2)c1=O

III. Inactive against both *T. vaginalis* and HeLa

**241**	<5.00	>10	<5.00	>10	ND	C[C@@H](NC(=O)C(Cc1ccccc1)N=[N+]=[N-])C(=O)N[C@@H](Cc1c[nH]c2ccccc12)C(=O)N[C@@H](CC1CCCCC1)C(=O)[C@@]1(C)CO1

**242**	<5.00	>10	<5.00	>10	ND	CC(C)C[C@H](NC(=O)[C@@H](NC(=O)N[C@@H](C(C)C)C(O)=O)C(C)C)C(=O)N[C@@H](CC(C)C)C(=O)[C@@]1(C)CO1
**243**	<5.00	>10	<5.00	>10	ND	[H][C@]12CCCC[C@@]1([H])[C@H](C[C@H](NC(=O)[C@H](Cc1ccc(OC)cc1)NC(=O)[C@H](C)NC(=O)CN1CCOCC1)C(=O)[C@@]1(C)CO1)CCC2
**244**	<5.00	>10	<5.00	>10	ND	OC(=O)C(F)(F)F.COc1ccc(C[C@H](NC(=O)[C@H](C)NC(=O)CN2CCOCC2)C(=O)N[C@@H](CC2CCC(F)(F)CC2)C(=O)[C@@]2(C)CO2)cc1
**245**	<5.00	>10	<5.00	>10	ND	COc1ccc(C[C@H](NC(=O)[C@H](C)NC(=O)CN2CCOCC2)C(=O)N[C@@H](CC2CCC(C)CC2)C(=O)[C@]2(C)CO2)cc1
**246**	<5.00	>10	<5.00	>10	ND	O=C(N[C@@H](C(C)C)C(N[C@@H](CCS(CCN)(=O)=O)C(N[C@@H](CC(C)C)C([C@]1(CO1)C)=O)=O)=O)CCCCC
**247**	<5.00	>10	<5.00	>10	ND	CC(C)C[C@H](NC(=O)[C@@H](NC(=O)N[C@@H](C(C)C)C(=O)NCc1ccccc1)C(C)C)C(=O)N[C@@H](CC1CCCCC1)C(=O)[C@@]1(C)CO1
**248**	<5.00	>10	<5.00	>10	ND	C[C@@]1(CO1)C(=O)[C@H](Cc1ccccc1)NC(=O)[C@H](Cc1ccccc1)NC(=O)CNC(=O)CN1CCOCC1
**249**	<5.00	>10	<5.00	>10	ND	CC(C)[C@H](NC(=O)N[C@@H](CO)C(=O)NCc1cccnc1)C(=O)N[C@@H](Cc1ccncc1)C(=O)N[C@@H](Cc1ccccc1)C(=O)[C@@]1(C)CO1
**250**	<5.00	>10	<5.00	>10	ND	CC(C)[C@H](NC(=O)N[C@@H](CO)C(=O)NCc1cccnc1)C(=O)N[C@@H](Cc1ccncc1)C(=O)N[C@@H](CC1CCCCC1)C(=O)[C@@]1(C)CO1
**251**	<5.00	>10	<5.00	>10	ND	C[C@@]1(CO1)C(=O)[C@H](CCc1ccccc1)NC(=O)[C@H](Cc1ccccc1)NC(=O)CNC(=O)CN1CCOCC1
**252**	<5.00	>10	<5.00	>10	ND	OC(=O)C(F)(F)F.C[C@@H](NC(=O)CN1CCOCC1)C(=O)N[C@@H](Cc1ccc(OCCN=[N+]=[N-])cc1)C(=O)N[C@@H](CC1CCCCC1)C(=O)[C@@]1(C)CO1
**253**	<5.00	>10	<5.00	>10	ND	C[C@H](NC(=O)CN1CCOCC1)C(=O)N[C@@H](Cc1ccc(OCc2cn(CNC(=O)C3CC4CC3C=C4)nn2)cc1)C(=O)N[C@@H](CC1CCCC2CCCCC12)C(=O)[C@@]1(C)CO1
**254**	<5.00	>10	<5.00	>10	ND	CCCC[C@H](NC(=O)[C@@H]1C[C@H](F)CN1C(=O)[C@H](C)NC(=O)CN=[N+]=[N-
**255**	<5.00	>10	<5.00	>10	ND	CCCC[C@H](NC(=O)[C@@H]1C[C@@H](F)CN1C(=O)[C@H](C)NC(=O)CN=[N+]=[N-])C(=O)N[C@@H](CC(C)C)C(=O)[C@@]1(C)CO1
**256**	<5.00	>10	<5.00	>10	ND	CCCC[C@H](NC(=O)[C@@H]1CCCN1C(=O)[C@H](C)NC(=O)[C@H](Cc1ccc(nc1)-c1ccccn1)NC(C)=O)C(=O)N[C@@H](CC(C)C)C(=O)[C@@]1(C)CO1

**257**	<5.00	>10	<5.00	>10	ND	CC(C)C[C@H](NC(=O)[C@H](C)NC(=O)[C@H](Cc1ccccc1)N=[N+]=[N-])C(=O)N[C@@H](CC12CC3CC(CC(C3)C1)C2)C(=O)[C@@]1(C)CO1
**258**	<5.00	>10	<5.00	>10	ND	CC(C)C[C@H](NC(=O)[C@H](CC12CC3CC(CC(C3)C1)C2)NC(=O)[C@H](Cc1ccccc1)N=[N+]=[N-])C(=O)N[C@@H](C)C(=O)[C@@]1(C)CO1
**259**	<5.00	>10	<5.00	>10	ND	CC(C)C[C@H](NC(=O)[C@H](CC(C)C)NC(=O)[C@H](Cc1ccccc1)N=[N+]=[N-])C(=O)N[C@@H](CC12CC3CC(CC(C3)C1)C2)C(=O)[C@@]1(C)CO1
**260**	<5.00	>10	<5.00	>10	ND	COc1ccc(C[C@H](NC(=O)[C@H](C)NC(=O)CN2CCOCC2)C(=O)N[C@@H](CC23CC4CC(CC(C4)C2)C3)C(=O)[C@@]2(C)CO2)cc
**261**	<5.00	>10	<5.00	>10	ND	COc1ccc(C[C@H](NC(=O)[C@H](CC23CC4CC(CC(C4)C2)C3)NC(=O)CN2CCOCC2)C(=O)N[C@@H](C)C(=O)[C@@]2(C)CO2)cc1
**262**	<5.00	>10	<5.00	>10	ND	OC(=O)C(F)(F)F.COc1ccc(C[C@H](NC(=O)[C@H](C)NC(=O)CN2CCOCC2)C(=O)N[C@@H](Cc2cccc3ccccc23)C(=O)[C@@]2(C)CO2)cc1
**263**	<5.00	>10	<5.00	>10	ND	CCCC[C@H](NC(=O)C1CCCN1C(=O)[C@H](C)NC(=O)CN=[N+]=[N-])C(=O)N[C@@H](C)C(=O)[C@@]1(C)CO1
**264**	<5.00	>10	<5.00	>10	ND	CCCC[C@H](NC(=O)C1CCCN1C(=O)[C@H](C)NC(=O)CN=[N+]=[N-])C(=O)N[C@@H](C(C)C)C(=O)[C@@]1(C)CO1
**265**	<5.00	>10	<5.00	>10	ND	C[C@H](NC(=O)[C@H](Cc1ccccc1)n1cc(CCCCC2=C3C(C)=CC(C)=[N]3[B-](F)(F)n3c(C)cc(C)c23)nn1)C(=O)N[C@@H](Cc1c[nH]c2ccccc12)C(=O)N[C@@H](CC1CCCCC1)C(=O)[C@@]1(C)CO1
**266**	<5.00	>10	<5.00	>10	ND	C[C@@H](NC(=O)CN=[N+]=[N-])C(=O)N[C@@H](Cc1c[nH]c2ccccc12)C(=O)N[C@@H](CC1CCCCC1)C(=O)[C@@]1(C)CO1
**267**	<5.00	>10	<5.00	>10	ND	CCCC[C@H](NC(=O)[C@@H]1CCCCN1C(=O)[C@H](C)NC(=O)CN=[N+]=[N-])C(=O)N[C@@H](CC(C)C)C(=O)[C@@]1(C)CO1
**268**	<5.00	>10	<5.00	>10	ND	CC[C@H](C)C(NC(=O)CCCCCNC(=O)CCCCCNC(=O)CCCCCNC(=O)CC12CC3C[C@@H](CC(C3)C1)C2)C(=O)N[C@@H]([C@@H](C)CC)C(=O)NC(C(C)O)C(=O)N[C@@H](CC(C)C)B1OC2[C@@H]3C[C@H](C[C@]2(C)O1)C3(C)C
**269**	<5.00	>10	<5.00	>10	ND	O=C([C@H](CCCC)NC([C@H](NC(CCCCC)=O)C(C)C)=O)N[C@@H](CC1=CC=CC=C1)C(C(C)=C)=O
**270**	<5.00	>10	<5.00	>10	ND	C[C@@]1(C([C@@H](NC([C@H](CCCC)NC([C@H](NC(CCCCC)=O)CCC(O)=O)=O)=O)CC2=CC=CC=C2)=O)OC1
**271**	<5.00	>10	<5.00	>10	ND	CCCC[C@H](NC(=O)[C@@H]1CSCN1C(=O)[C@H](C)NC(=O)CN=[N+]=[N-])C(=O)N[C@@H](CC(C)C)C(=O)[C@@]1(C)CO1

**272**	<5.00	>10	<5.00	>10	ND	CCCC[C@H](NC(=O)[C@@H]1CC(F)(F)CN1C(=O)[C@H](C)NC(=O)CN=[N+]=[N-])C(=O)N[C@@H](CC(C)C)C(=O)[C@@]1(C)CO1
**273**	<5.00	>10	<5.00	>10	ND	CC[C@H](C)[C@H](NC(=O)Cn1cc(CNC(=O)C2CC3CC2C=C3)nn1)C(=O)N[C@@H]([C@@H](C)CC)C(=O)N[C@@H]([C@@H](C)O)C(=O)N[C@@H](CC(C)C)C(=O)[C@@]1(C)CO1
**274**	<5.00	>10	<5.00	>10	ND	CC[C@H](C)[C@H](NC(=O)Cn1cc(CCCCC2=C3C(C)=CC(C)=[N]3[B-](F)(F)n3c(C)cc(C)c23)nn1)C(=O)N[C@@H]([C@@H](C)CC)C(=O)N[C@@H]([C@@H](C)O)C(=O)N[C@@H](CC(C)C)C(=O)[C@@]1(C)CO1
**275**	<5.00	>10	<5.00	>10	ND	O=C(N[C@@H](CC1=C(F)C=C(F)C=C1)C([C@]2(C)OC2)=O)[C@H](CC3=CC(OCC4=CN(N=N4)CC(NCC[C@@H]5NC(CCCCC)=O)=O)=CC=C3)NC5=O
**276**	<5.00	>10	<5.00	>10	ND	CC(C)CC(NC(=O)C(NC(=O)C(CO)NC(=O)C(CO)NC(=O)CN=[N+]=[N-])C(C)O)C(=O)C1(C)CO1
**277**	<5.00	>10	<5.00	>10	ND	CC(C)C[C@@H](NC(=O)COCCOCCOCc1cn(CC(=O)N[C@@H](CSSC(C)(C)C)C(=O)N[C@@H](CSSC(C)(C)C)C(=O)N(C)C)nn1)C(=O)N[C@H](CC(C)C)C(=O)N[C@H](CC(C)C)C(=O)c1ccco1
**278**	<5.00	>10	<5.00	>10	ND	CC(C)CC(NC(=O)[C@H](Cc1ccccc1)NC(=O)c1cnccn1)C(=O)[C@@]1(C)CO1
**279**	<5.00	>10	<5.00	>10	ND	CC(C)C[C@H](NC(=O)[C@H](CC(C)C)NC(=O)[C@H](CC(C)C)NC(=O)CCCCCNC(=O)CCCCCNC(=O)CCCCCNC(=O)[C@H](CCCCNC(=O)CCCCC1SCC2NC(=O)NC12)NC(=O)CC12CC3C[C@@H](CC(C3)C1)C2)\C=C\S(C)(=O)=O
**280**	<5.00	>10	<5.00	>10	ND	CCCC[C@H](NC(=O)[C@@H]1CCCN1C(=O)[C@H](C)NC(C)=O)C(=O)N[C@@H](CC(C)C)C(=O)[C@@]1(C)CO1
**281**	<5.00	>10	<5.00	>10	ND	CC(C)C[C@H](NC(=O)[C@H](Cc1c(F)c(F)c(F)c(F)c1F)NC(=O)[C@H](Cc1ccccc1)NC(=O)[C@H](Cc1ccccc1)n1cc(CCCCC2=C3C(C)=CC(C)=[N]3[B-](F)(F)n3c(C)cc(C)c23)nn1)C(=O)[C@@]1(C)CO1
**282**	<5.00	>10	<5.00	>10	ND	[Cl-].CC(C)C[C@H](NC(=O)OCc1ccccc1)C(=O)N[C@@H](CC(C)C)C(=O)N[C@@H](CCCC[NH3+])\C=C\S(C)(=O)=O
**283**	<5.00	>10	<5.00	>10	ND	CC(C)C[C@H](NC(=O)[C@H](CCCNC(N)=N)NC(=O)[C@H](Cc1ccc(O)cc1)NC(C)=O)C(=O)NC(CCCCN)\C=C\S(C)(=O)=O
**284**	<5.00	>10	<5.00	>10	ND	CC(C)C[C@H](NN[C@@H](CC(C)C)C1=CC(=O)OC1)C(=O)N[C@@H](CC(C)C)C(=O)N[C@H](Cc1ccccc1)C(=O)N=[N+]=[N-]
**285**	<5.00	>10	<5.00	>10	ND	CCOP(=O)(OCC)\C=C\[C@H](CC(C)C)NN[C@@H](CC(C)C)C(=O)N[C@@H](CC(C)C)C(=O)N[C@H](Cc1ccccc1)C(=O)N=[N+]=[N-]

**286**	<5.00	>10	<5.00	>10	ND	FC(F)(F)C=O.CC(C)C[C@H](NC(=O)[C@H](CC(C)C)NC(=O)[C@H](Cc1ccccc1)N=[N+]=[N-])C(=O)N[C@@H](CCCCN)\C=C\S(C)(=O)=O
**287**	<5.00	>10	<5.00	>10	ND	FC(F)(F)C=O.CC(C)C[C@H](NC(=O)[C@H](CC(C)C)NC(=O)[C@H](Cc1ccccc1)N=[N+]=[N-
**288**	<5.00	>10	<5.00	>10	ND	C\C=C\C(=O)[C@H](CC(C)C)NN[C@@H](CC(C)C)C(=O)N[C@@H](CC(C)C)C(=O)N[C@H](Cc1ccccc1)C(=O)N=[N+]=[N-]
**289**	<5.00	>10	<5.00	>10	ND	FC(F)(F)C=O.CC(C)C[C@H](NC(=O)[C@H](CC(C)C)NC(=O)[C@H](Cc1ccccc1)N=[N+]=[N-])C(=O)N[C@@H](CCCN)\C=C\S(C)(=O)=O
**290**	<5.00	>10	<5.00	>10	ND	FC(F)(F)C=O.CC(C)C[C@H](NC(=O)[C@H](Cc1ccccc1)N=[N+]=[N-])C(=O)N[C@@H](C)C(=O)N[C@@H](CCN)\C=C\S(C)(=O)=O
**291**	<5.00	>10	<5.00	>10	ND	FC(F)(F)C=O.CC(C)C[C@H](NC(=O)[C@H](CC(C)C)NC(=O)[C@H](CCCN)NC(=O)[C@H](Cc1ccccc1)N=[N+]=[N-
**292**	<5.00	>10	<5.00	>10	ND	CC(C)C[C@H](NC(=O)[C@H](CC(C)C)NC(=O)c1cccc(NC(=O)Nc2ccccn2)n1)C(=O)N[C@@H](CC(C)C)C(=O)c1ccco1
**293**	<5.00	>10	<5.00	>10	ND	CC(C)C[C@H](NC(=O)CCCCC(=O)Nc1ccc2ccc(NC(C)=O)nc2n1)C(=O)N[C@@H](CC(C)C)C(=O)N[C@@H](CC(C)C)C(=O)c1ccco
**294**	<5.00	>10	<5.00	>10	ND	FC(F)(F)C=O.CC(C)C[C@H](NC(=O)[C@H](CC(C)C)NC(=O)[C@H](CN)NC(=O)[C@H](Cc1ccccc1)N=[N+]=[N-])\C=C\S(C)(=O)=O
**295**	<5.00	>10	<5.00	>10	ND	OC(=O)C(F)(F)F.CC(C)C[C@H](NC(=O)[C@H](Cc1ccccc1)NC(=O)CN=[N+]=[N-])C(=O)N[C@@H](C)C(=O)N[C@@H](Cc1ccc(CN)cc1)\C=C\S(C)(=O)=O
**296**	<5.00	>10	<5.00	>10	ND	FC(F)(F)C=O.CC(C)C[C@H](NC(=O)[C@H](Cc1ccccc1)N=[N+]=[N-])C(=O)NCC(=O)N[C@@H](Cc1ccc(CN)cc1)\C=C\S(C)(=O)=O
**297**	<5.00	>10	<5.00	>10	ND	CC(C)C[C@H](NC(=O)[C@@H](NC(=O)N[C@H](C(C)C)C(O)=O)C(C)C)C(=O)[C@@]1(C)CO1
**298**	<5.00	>10	<5.00	>10	ND	CCCCCCCCCCNC(=O)N[C@@H](C(C)C)C(=O)N[C@@H](CC(C)C)C(=O)[C@@]1(C)CO1
**299**	<5.00	>10	<5.00	>10	ND	OC(=O)C(F)(F)F.CC(C)C[C@H](NC(=O)[C@H](CC(C)C)NC(=O)[C@H](Cc1ccccc1)N=[N+]=[N-])C(=O)N[C@@H](Cc1cnn[nH]1)\C=C\S(C)(=O)=O
**300**	<5.00	>10	<5.00	>10	ND	OC(=O)C(F)(F)F.OC(=O)C(F)(F)F.CC(C)C[C@H](NC(=O)[C@H](CCONC(N)=N)NC(=O)[C@H](Cc1ccccc1)N=[N+]=[N-])C(=O)N[C@@H](Cc1ccc(CN)cc1)\C=C\S(C)(=O)=O
**301**	<5.00	>10	<5.00	>10	ND	CCCC[C@H](NC(=O)[C@@H]1CCCN1C(=O)[C@H](C)NC(=O)CN=[N+]=[N-])C(=O)N[C@@H](CCC(O)=O)C(=O)[C@@]1(C)CO1
**302**	<5.00	>10	<5.00	>10	ND	COc1ccc(C[C@H](NC(=O)[C@H](C)NC(=O)CN2CCOCC2)C(=O)N[C@@H](Cc2ccccc2)\C=C\S(C)(=O)=O)cc1

**303**	<5.00	>10	<5.00	>10	ND	C[C@@H](NC(=O)C1=C(C)c2ccccc2C1)C(=O)N[C@@H](Cc1c[nH]c2ccccc12)C(=O)N[C@@H](CC1CCCCC1)\C=C\S(C)(=O)=O
**304**	<5.00	>10	<5.00	>10	ND	C[C@@H](NC(=O)C1=C(C)c2ccccc2C1)C(=O)N[C@@H](Cc1c[nH]c2ccccc12)C(=O)N[C@@H](Cc1ccccc1)\C=C\S(C)(=O)=O
**305**	<5.00	>10	<5.00	>10	ND	CC(C)C[C@H](NC(=O)[C@H](CC(C)C)n1cc(C)cc(NC(=O)c2cc(C)on2)c1=O)C(=O)[C@@]1(C)CO1
**306**	<5.00	>10	<5.00	>10	ND	COc1ccc(C[C@H](NC(=O)[C@H](C)NC(=O)c2cnc(C)s2)C(=O)N[C@@H](CCC2CCCCC2)C(=O)[C@@]2(C)CO2)cc1
**307**	<5.00	>10	<5.00	>10	ND	CCC(C)[C@H](NC(C)=O)C(=O)N[C@@H](C(C)CC)C(=O)N[C@@H](CNC(=O)CCCC(=O)N1CC=C1)C(=O)N[C@@H](CC(C)C)C(=O)[C@@]1(C)CO1
**308**	<5.00	>10	<5.00	>10	ND	CC(C)CC(NC(=O)[C@H](Cc1ccccc1)NC(=O)c1cnccn1)\C=C\S(C)(=O)=O
**309**	<5.00	>10	<5.00	>10	ND	COc1ccc(C[C@H](NC(=O)CCC2=C(C)C3=Cc4ccc(-c5ccc(OCCCN=[N+]=[N-])cc5)n4[B-](F)(F)[N]3=C2C)C(=O)N[C@@H](Cc2ccccc2)C(=O)N[C@@H](CC(C)C)\C=C\S(C)(=O)=O)cc1
**310**	<5.00	>10	<5.00	>10	ND	CC(C)[C@H](NC(=O)c1cccc(O)c1C)C(=O)N[C@@H](CO)C(=O)N[C@@H](Cc1ccc(CN)cc1)\C=C\S(C)(=O)=O
**311**	<5.00	>10	<5.00	>10	ND	OC(=O)C(F)(F)F.COc1ccc(C[C@H](NC(=O)[C@H](C)NC(=O)CN2CCOCC2)C(=O)N[C@@H](C[C@@H]2CCCc3ccccc23)C(=O)[C@@]2(C)CO2)cc1
**312**	<5.00	>10	<5.00	>10	ND	C[C@H](NC(=O)[C@H](Cc1ccccc1)NC(=O)[C@H](C[C@H]1CC[C@H](CC1)c1ccccc1)NC(=O)[C@@H](Cc1ccccc1)N=[N+]=[N-])C(=O)[C@@]1(C)CO1
**313**	<5.00	>10	<5.00	>10	ND	CCCC[C@H](NC(=O)[C@@H]1CCCN1C(=O)[C@H](C)NC(=O)Cn1cc(CNC(=O)C2CC3CC2C=C3)nn1)C(=O)N[C@@H](CC(O)=O)C(=O)[C@@]1(C)CO1
**314**	<5.00	>10	<5.00	>10	ND	C[C@H](NC(=O)[C@H](Cc1ccccc1)NC(=O)[C@H](C[C@H]1CC[C@@H](CC1)C1CCCCC1)NC(=O)[C@H](Cc1ccccc1)NC(=O)CCN1CC=C1)C(=O)[C@@]1(C)CO1
**315**	<5.00	>10	<5.00	>10	ND	CC(C)C[C@H](NN[C@@H](CC(C)C)C(=O)N[C@@H](CC(C)C)C(=O)C(=O)OCc1ccccc1)\C=C\S(=O)(=O)CC12CC3CC(CC(C3)C1)C2
**316**	<5.00	>10	<5.00	>10	ND	CC(C)C[C@H](NN[C@@H](CC(C)C)C(=O)N[C@@H](CC(C)C)C(=O)N[C@H](Cc1ccccc1)C(=O)N=[N+]=[N-])\C=C\S(=O)(=O)CC12CC3CC(CC(C3)C1)C2
**317**	<5.00	>10	<5.00	>10	ND	CC(C)C[C@H](NC(=O)[C@H](CC(C)C)NC(=O)[C@H](Cc1ccccc1)N=[N+]=[N-])C(=O)N[C@@H](CC12CC3CC(CC(C3)C1)C2)\C=C\S(C)(=O)=O

**318**	<5.00	>10	<5.00	>10	ND	CC(C)C[C@H](NC(=O)CCCCCNC(=O)CCCCCNC(=O)CCCCCNC(=O)CC12CC3CC(CC(C3)C1)C2)C(=O)N[C@@H](CC(C)C)C(=O)N[C@@H](CC12CC3CC(CC(C3)C1)C2)\C=C\S(C)(=O)=O
**319**	<5.00	>10	<5.00	>10	ND	CC(C)C[C@@H](NC(=O)[C@@H](CC(C)C)NC(=O)[C@H](CC(C)C)NC(=O)OCc1ccccc1)\C=C\S(C)(=O)=O
**320**	<5.00	>10	<5.00	>10	ND	CC(C)C[C@H](NC(=O)OCc1ccccc1)C(=O)N[C@@H](CC(C)C)C(=O)N[C@@H](CC(C)C)C(=O)C(C)=C
**321**	<5.00	>10	<5.00	>10	ND	CC(C)C[C@H](NC(=O)OCc1ccccc1)C(=O)N[C@@H](CC(C)C)C(=O)N[C@@H](CC(C)C)C1(CO1)C(C)=O
**322**	<5.00	>10	<5.00	>10	ND	CC(C)C[C@H](NC(=O)OCc1ccccc1)C(=O)N[C@@H](CC(C)C)C(=O)N[C@@H](CC(C)C)[C@@]1(CO1)[C@@]1(C)CO1
**323**	<5.00	>10	<5.00	>10	ND	CC(C)C[C@H](NC(=O)[C@@H](NC(=O)N[C@H](C(C)C)C(=O)NCc1ccccc1)C(C)C)\C=C\S(C)(=O)=O
**324**	<5.00	>10	<5.00	>10	ND	COc1ccc(C[C@H](NC(=O)[C@H](C)NC(=O)CN2CCOCC2)C(=O)N[C@@H](CC2CCC3CCCCC3C2)C(=O)[C@]2(C)CO2)cc1
**325**	<5.00	>10	<5.00	>10	ND	CO[C@H]1CCC(C[C@H](NC(=O)[C@H](Cc2ccc(OC)cc2)NC(=O)[C@H](C)NC(=O)CN2CCOCC2)C(=O)[C@]2(C)CO2)CC1
**326**	<5.00	>10	<5.00	>10	ND	CC(C)C[C@H](NC(=O)[C@@H](NC(=O)N[C@@H](C(C)C)C(=O)N[C@@H](CC(C)C)C(=O)OC(C)(C)C)C(C)C)\C=C\S(C)(=O)=O
**327**	<5.00	>10	<5.00	>10	ND	CC(C)C[C@H](NC(=O)N[C@@H](C(C)C)C(=O)N[C@@H](CC(C)C)C(=O)N[C@@H](CC(C)C)C(=O)OC(C)(C)C)\C=C\S(C)(=O)=O
**328**	<5.00	>10	<5.00	>10	ND	CC(C)C[C@H](NC(=O)[C@H](Cc1ccc(CN)cc1)NC(=O)[C@H](Cc1ccccc1)N=[N+]=[N-])C(=O)N[C@@H](CC(N)=O)\C=C\S(C)(=O)=O
**329**	<5.00	>10	<5.00	>10	ND	CC(C)C[C@H](NC(=O)[C@H](Cc1ccc(CN)cc1)NC(=O)[C@H](Cc1ccccc1)N=[N+]=[N-])C(=O)N[C@@H](Cc1ccncc1)\C=C\S(C)(=O)=O
**330**	<5.00	>10	<5.00	>10	ND	FC(F)(F)C=O.CC(C)C[C@H](NC(=O)c1cnccn1)C(=O)N[C@@H](C)C(=O)N[C@@H](Cc1ccc(CN)cc1)\C=C\S(C)(=O)=O
**331**	<5.00	>10	<5.00	>10	ND	CC(C)C[C@H](NC(=O)[C@H](Cc1ccccc1)N=[N+]=[N-])C(=O)N(C)[C@@H](CC(C)C)C(=O)N[C@@H](Cc1ccc(CN)cc1)\C=C\S(C)(=O)=O
**332**	<5.00	>10	<5.00	>10	ND	FC(F)(F)C=O.CC(C)C[C@H](NC(=O)[C@H](Cc1ccccc1)N=[N+]=[N-])C(=O)N[C@@H](C(C)O)C(=O)N[C@@H](Cc1ccc(CN)cc1)\C=C\
**333**	<5.00	>10	<5.00	>10	ND	CC(C)C[C@H](NC(=O)[C@H](CC(C)C)n1cc(C)cc(NC(=O)OCc2ccccc2)c1=O)C(=O)[C@@]1(C)CO1
**334**	<5.00	>10	<5.00	>10	ND	CC(C)C[C@H](NC(=O)[C@@H](CC(C)C)n1cc(C)cc(NC(=O)OCc2ccccc2)c1=O)C(=O)[C@@]1(C)CO1
**335**	<5.00	>10	<5.00	>10	ND	CC(C)C[C@H](NC(=O)C(CC(C)C)n1cc(C)cc(NC(=O)c2cc(Cl)ccc2Cl)c1=O)C(=O)[C@@]1(C)CO1

**336**	<5.00	>10	<5.00	>10	ND	CC(C)C[C@H](NC(=O)[C@@H](CC(C)C)n1cc(C)cc(NC(=O)c2cnccn2)c1=O)C(=O)[C@@]1(C)CO1
**337**	<5.00	>10	<5.00	>10	ND	CC(C)C[C@H](NC(=O)[C@@H](CC(C)C)n1cc(C)cc(NC(=O)c2cc(C)on2)c1=O)C(=O)[C@@]1(C)CO1
**338**	<5.00	>10	<5.00	>10	ND	CC(C)C[C@H](NC(=O)[C@H](CC(C)C)n1cc(C)cc(NC(=O)[C@H](Cc2ccccc2)N=[N+]=[N-])c1=O)C(=O)[C@@]1(C)CO1
**339**	<5.00	>10	<5.00	>10	ND	CC(C)C[C@H](NC(=O)[C@@H](CC(C)C)n1cc(C)cc(NC(=O)[C@H](Cc2ccccc2)N=[N+]=[N-])c1=O)C(=O)[C@@]1(C)CO1
**340**	<5.00	>10	<5.00	>10	ND	FC(F)(F)C=O.CC(C)C[C@H](NC(=O)[C@H](Cc1ccccc1)N=[N+]=[N-])C(=O)N[C@@H](CC(F)(F)F)C(=O)N[C@@H](Cc1ccc(CN)cc1)\C
**341**	<5.00	>10	<5.00	>10	ND	FC(F)(F)C=O.COC[C@H](NC(=O)[C@H](CC(C)C)NC(=O)[C@H](Cc1ccccc1)N=[N+]=[N-])C(=O)N[C@@H](Cc1ccc(CN)cc1)\C=C\S(C)(=O)=O
**342**	<5.00	>10	<5.00	>10	ND	FC(F)(F)C=O.COC[C@H](NC(=O)[C@H](CC(C)C)NC(=O)c1cnc(C)s1)C(=O)N[C@@H](Cc1ccc(CN)cc1)\C=C\S(C)(=O)=O
**343**	<5.00	>10	<5.00	>10	ND	CC(C)C[C@H](NC(=O)C(CC(C)C)n1cc(C)cc(NC(=O)OCc2ccccc2)c1=O)\C=C\S(C)(=O)=O
**344**	<5.00	>10	<5.00	>10	ND	CC(C)C[C@H](NC(=O)C(CC(C)C)n1cc(C)cc(NC(=O)c2cc(Cl)ccc2Cl)c1=O)\C=C\S(C)(=O)=O
**345**	<5.00	>10	<5.00	>10	ND	OC(=O)C(F)(F)F.CC(C)CC(C(=O)N[C@@H](Cc1ccc(CN)cc1)\C=C\S(C)(=O)=O)n1cc(C)cc(NC(=O)c2cc(Cl)ccc2Cl)c1=O
**346**	<5.00	>10	<5.00	>10	ND	OC(=O)C(F)(F)F.CC(C)CC(C(=O)N[C@@H](Cc1ccc(CN)cc1)\C=C\S(C)(=O)=O)n1cc(C)cc(NC(=O)c2cnccn2)c1=O
**347**	<5.00	>10	<5.00	>10	ND	CC(C)C[C@H](NC(=O)[C@@H](CC(C)C)n1cc(C)cc(NC(=O)c2cc(C)on2)c1=O)\C=C\S(C)(=O)=O
**348**	<5.00	>10	<5.00	>10	ND	OC(=O)C(F)(F)F.OC(=O)C(F)(F)F.CC(C)[C@H](NC(=O)OCc1ccccc1)C(=O)N[C@@H](CCCNC(N)=N)C(=O)N1CCC[C@H]1C(=O)N[C@@H](CCCNC(N)=N)\C=C\S(C)(=O)=O
**349**	<5.00	>10	<5.00	>10	ND	OC(=O)C(F)(F)F.CC(C)C[C@H](NC(=O)c1cnc(C)s1)C(=O)N[C@@H](CCC1CCCCC1)C(=O)N[C@@H](Cc1ccc(CN)cc1)\C=C\S(C)(=O)=O
**350**	<5.00	>10	<5.00	>10	ND	OC(=O)C(F)(F)F.CC(C)C[C@H](NC(=O)[C@H](CC1CCCCC1)NC(=O)c1cnc(C)s1)C(=O)N[C@@H](Cc1ccc(CN)cc1)\C=C\S(C)(=O)=O
**351**	<5.00	>10	<5.00	>10	ND	OC(=O)C(F)(F)F.CC(C)C[C@H](NC(=O)c1cnc(C)s1)C(=O)N[C@@H](CC1CCCCC1)C(=O)N[C@@H](Cc1ccc(CN)cc1)\C=C\S(C)(=O)=O
**352**	<5.00	>10	<5.00	>10	ND	OC(=O)C(F)(F)F.COC1CCC(C[C@H](NC(=O)[C@H](CC(C)C)NC(=O)c2cnc(C)s2)C(=O)N[C@@H](Cc2ccc(CN)cc2)\C=C\S(C)(=O)=O)CC1

**353**	<5.00	>10	<5.00	>10	ND	OC(=O)C(F)(F)F.CC(C)C[C@H](NC(=O)[C@H](CC1CCC(C)CC1)NC(=O)c1cnc(C)s1)C(=O)N[C@@H](Cc1ccc(CN)cc1)\C=C\S(C)(=O)=O
**354**	<5.00	>10	<5.00	>10	ND	OC(=O)C(F)(F)F.CC(C)C[C@H](NC(=O)[C@H](CCC1CCCCC1)NC(=O)c1cnc(C)s1)C(=O)N[C@@H](Cc1ccc(CN)cc1)\C=C\S(C)(=O)=O
**355**	<5.00	>10	<5.00	>10	ND	OC(=O)C(F)(F)F.CC(C)C[C@H](NC(=O)[C@@H](NC(=O)c1cnc(C)s1)C1CCCCC1)C(=O)N[C@@H](Cc1ccc(CN)cc1)\C=C\S(C)(=O)=O
**356**	<5.00	>10	<5.00	>10	ND	COc1ccc(C[C@H](NC(=O)[C@H](C)NC(=O)CN2CCOCC2)C(=O)N[C@@H](C2CCCCC2)C(=O)[C@@]2(C)CO2)cc1
**357**	<5.00	>10	<5.00	>10	ND	COc1ccc(C[C@H](NC(=O)[C@H](C)NC(=O)CN2CCOCC2)C(=O)N[C@@H](CCC2CCCCC2)C(=O)[C@@]2(C)CO2)cc1
**358**	<5.00	>10	<5.00	>10	ND	COc1ccc(C[C@H](NC(=O)[C@H](C)NC(=O)CN2CCOCC2)C(=O)N[C@@H](CC2CCCC3CCCCC23)C(=O)[C@@]2(C)CO2)cc1
**359**	<5.00	>10	<5.00	>10	ND	OC(=O)C(F)(F)F.CC(C)C[C@H](NC(=O)[C@H](CC1CCC(CC1)C1CCCCC1)NC(=O)c1cnc(C)s1)C(=O)N[C@@H](Cc1ccc(CN)cc1)\C=C\S(C)(=O)=O
**360**	<5.00	>10	<5.00	>10	ND	COc1ccc(C[C@H](NC(=O)[C@H](C)NC(=O)CN2CCOCC2)C(=O)N[C@@H](CC2CCC(CC2)C(F)(F)F)C(=O)[C@@]2(C)CO2)cc1
**361**	<5.00	>10	<5.00	>10	ND	OC(=O)C(F)(F)F.CC(C)C[C@H](NC(=O)[C@H](CC1CCCC2CCCCC12)NC(=O)c1cnc(C)s1)C(=O)N[C@@H](Cc1ccc(CN)cc1)\C=C\S(C)(=O)=O
**362**	<5.00	>10	<5.00	>10	ND	OC(=O)C(F)(F)F.CC(C)C[C@H](NC(=O)c1cnc(C)s1)C(=O)N[C@@H](CC1CCCC2CCCCC12)C(=O)N[C@@H](Cc1ccc(CN)cc1)\C=C\S(C)(=O)=O
**363**	<5.00	>10	<5.00	>10	ND	OC(=O)C(F)(F)F.Cc1ncc(s1)C(=O)N[C@@H](CC1CCCCC1)C(=O)N[C@@H](CC1CCCCC1)C(=O)N[C@@H](Cc1ccc(CN)cc1)\C=C\S(C)(=O)=O
**364**	<5.00	>10	<5.00	>10	ND	OC(=O)C(F)(F)F.[H][C@]12CCCC[C@@]1([H])[C@@H](C[C@H](NC(=O)[C@H](Cc1ccc(OC)cc1)NC(=O)[C@H](C)NC(=O)CN1CCOCC1)C(=O)[C@@]1(C)CO1)CCC2
**365**	<5.00	>10	<5.00	>10	ND	FC(F)(F)C=O.CC(C)C[C@H](NC(=O)[C@H](CC(C)C)NC(=O)c1cnccn1)C(=O)N[C@@H](Cc1ccc(CN)cc1)\C=C\S(C)(=O)=O
**366**	<5.00	>10	<5.00	>10	ND	FC(F)(F)C=O.CC(C)C[C@H](NC(=O)[C@H](CC(C)C)NC(=O)c1ccccc1)C(=O)N[C@@H](Cc1ccc(CN)cc1)\C=C\S(C)(=O)=O
**367**	<5.00	>10	<5.00	>10	ND	FC(F)(F)C=O.FC(F)(F)C=O.CC(C)C[C@H](NC(=O)CN1CCOCC1)C(=O)N[C@@H](CC(C)C)C(=O)N[C@@H](Cc1ccc(CN)cc1)\C=C\S(C)(=O)=O
**368**	<5.00	>10	<5.00	>10	ND	CCCC[C@H](NC(=O)[C@@H]1CC(F)(F)CN1C(=O)[C@H](C)NC(=O)Cn1cc(CNC(=O)C2CC3CC2C=C3)nn1)C(=O)N[C@@H](CC1CCCCC1)C(=O)[C@@]1(C)CO1

**369**	<5.00	>10	<5.00	>10	ND	FC(F)(F)C=O.CC(C)C[C@H](NC(=O)[C@H](CC(C)C)NC(=O)[C@H](Cc1ccccc1)NC(=O)CN=[N+]=[N-])C(=O)N[C@@H](Cc1ccc(CN)cc1)\C=C\S(C)(=O)=O
**370**	<5.00	>10	<5.00	>10	ND	OC(=O)C(F)(F)F.CC(C)C[C@H](NC(=O)[C@H](Cc1ccccc1)n1cc(CNC(=O)C2CC3CC2C=C3)nn1)C(=O)N[C@@H](C)C(=O)N[C@@H](Cc1ccc(CN)cc1)\C=C\S(C)(=O)=O
**371**	<5.00	>10	<5.00	>10	ND	FC(F)(F)C=O.CC(C)C[C@H](NC(=O)[C@H](CC(C)C)NC(=O)c1cc(Cl)ccc1Cl)C(=O)N[C@@H](Cc1ccc(CN)cc1)\C=C\S(C)(=O)=O
**372**	<5.00	>10	<5.00	>10	ND	OC(=O)C(F)(F)F.CC(C)C[C@H](NC(=O)[C@H](Cc1ccccc1)N=[N+]=[N-])C(=O)NC[C@@H](C)C(=O)N[C@@H](Cc1ccc(CN)cc1)\C=C\S(C)(=O)=O
**373**	<5.00	>10	<5.00	>10	ND	FC(F)(F)C=O.CC(C)C[C@H](NC(=O)[C@H](CC(C)C)NC(=O)c1ccc(nc1)-c1ccccc1)C(=O)N[C@@H](Cc1ccc(CN)cc1)\C=C\S(C)(=O)=O

The indicated proteasome inhibitors were tested for inhibitory activity (50% growth inhibitory concentration, GI50) against *T. vaginalis* F1623 and human HeLa cells in 24-48 h growth and survival assays. Data are mean and SE of pGI50 values obtainbed from at least three independent experiments. GI50 values were calculated from the mean pGI50. The selectivity index was calculated as GI50 in HeLa cells over GI50 in *T. vaginalis*. Entries are listed in three groups: I. Compounds active (GI50<10 µM) against *T. vaginalis*, ordered by decreasing pGI50 against *T. vaginalis*; II. Compounds inactive (GI50 >10 µM) against *T. vaginalis* but active (GI50<10 µM) against HeLa, ordered by decreasing GI50 against HeLa; III. Compounds inactive (GI50 >10 µM) against both *T. vaginalis* and HeLa. ND - Not determined.

Supplemental Table 2. Predicted ADME properties of top proteasome inhibitors

Descriptor	Description	Recommendations	Cpd 8	Cpd 12	Metronidazole	Tinidazole	Paromomycin
Human Oral Absorption (%)	Predicted human oral absorption on 0 to 100% scale, based on a quantitative multiple linear regression model	>80% high <25% poor	71.6	80.5	70.0	70.2	0
QPPCaco	Predicted apparent Caco-2 cell permeability in nm/sec.[a]	<25 poor >500 great	193.3	594.2	258.3	235.0	0
QPPMDCK	Predicted apparent MDCK cell permeability in nm/sec.[a]	<25 poor >500 great	238.4	488.7	114.5	103.4	0
QPlogBB	Predicted brain/blood partition coefficient	−3.0 to 1.2	-1.97	-1.47	-0.96	-1.23	-4.77
QPlogHERG	Predicted IC_{50} value for blockage of HERG K+ channels	>−5	-1.45	-4.71	-2.93	-3.81	-8.15
QPlogKhsa	Prediction of binding to human serum albumin	−1.5 to 1.5	-0.67	0.26	-0.69	-1.05	-2.16
QPlogKp	Predicted skin permeability	−8.0 to −1.0	-1.82	-0.51	-4.01	-4.00	-12.36
QPlogS	Predicted aqueous solubility	−6.5 to 0.5	-3.24	-6.18	-1.25	-0.90	2.00
RuleOfFive	Number of violations of Lipinski's rule of five (MW <500, QPlogPo/w <5, donorHB ≤ 5, accptHB ≤10)	Maximum 4, Fewer violations→better drug likeness	1	2	0	0	3
RuleOfThree	Number of violations of Jorgensen's rule of three (QPlogS >-5.7, QP PCaco >22 nm/s, primary metabolites <7)	Maximum 3, Fewer violations→better drug likeness	0	2	0	0	2
MW	Molecular weight	130 to 725	503.6	587.7	171.2	247.3	615.6

ADME predictions for two *T. vaginalis* selective proteasome inhibitors (compounds **8** and **12**), two structurally unrelated control drugs commonly used in the treatment of trichomoniasis (metronidazole and tinidazole), and a broad-spectrum antibiotic with poor oral absorption (paromomycin) were generated using Schroedinger QikProp starting from compound conformations generated by ligprep.[a]Predictions are for non-active transport.
